# H3K27 modifiers regulate lifespan in *C. elegans* in a context-dependent manner

**DOI:** 10.1186/s12915-021-00984-8

**Published:** 2021-03-25

**Authors:** Abigail R. R. Guillermo, Karolina Chocian, Gavriil Gavriilidis, Julien Vandamme, Anna Elisabetta Salcini, Jane Mellor, Alison Woollard

**Affiliations:** 1 Department of Biochemistry, University of Oxford, Oxford, UK; 2 Present Address: Department of Physiology, National University of Singapore, Singapore, Singapore; 3 Biotech Research and Innovation Centre (BRIC), University of Copenhagen, Copenhagen, Denmark; 4 Present Address: Department of Health Technology, Technical University of Denmark, Kongens Lyngby, Denmark

**Keywords:** Ageing, Lifespan, Healthspan, *C. elegans*, Chromatin, Histone demethylase, Histone methyltransferase, H3K27

## Abstract

**Background:**

Evidence of global heterochromatin decay and aberrant gene expression in models of physiological and premature ageing have long supported the “heterochromatin loss theory of ageing”, which proposes that ageing is aetiologically linked to, and accompanied by, a progressive, generalised loss of repressive epigenetic signatures. However, the remarkable plasticity of chromatin conformation suggests that the re-establishment of such marks could potentially revert the transcriptomic architecture of animal cells to a “younger” state, promoting longevity and healthspan. To expand our understanding of the ageing process and its connection to chromatin biology, we screened an RNAi library of chromatin-associated factors for increased longevity phenotypes.

**Results:**

We identified the lysine demethylases *jmjd-3.2* and *utx-1*, as well as the lysine methyltransferase *mes-2* as regulators of both lifespan and healthspan in *C. elegans*. Strikingly, we found that both overexpression and loss of function of *jmjd-3.2* and *utx-1* are all associated with enhanced longevity. Furthermore, we showed that the catalytic activity of UTX-1, but not JMJD-3.2, is critical for lifespan extension in the context of overexpression. In attempting to reconcile the improved longevity associated with both loss and gain of function of *utx-1*, we investigated the alternative lifespan pathways and tissue specificity of longevity outcomes. We demonstrated that lifespan extension caused by loss of *utx-1* function is *daf-16* dependent, while overexpression effects are partially independent of *daf-16*. In addition, lifespan extension was observed when *utx-1* was knocked down or overexpressed in neurons and intestine, whereas in the epidermis, only knockdown of *utx-1* conferred improved longevity.

**Conclusions:**

We show that the regulation of longevity by chromatin modifiers can be the result of the interaction between distinct factors, such as the level and tissue of expression. Overall, we suggest that the heterochromatin loss model of ageing may be too simplistic an explanation of organismal ageing when molecular and tissue-specific effects are taken into account.

**Supplementary Information:**

The online version contains supplementary material available at 10.1186/s12915-021-00984-8.

## Background

Despite the seemingly multifactorial nature of the ageing process, single genes have been identified which exert a disproportionate effect on longevity. For example, interference with the insulin signalling pathway, through mutation of the insulin-like growth factor receptor *daf-2*/IGF1, has a profound lifespan-extending effect in several organisms [1–3]. Not only is lifespan extended in such mutants, but later-life health is often improved as well [4, 5] suggesting that increased understanding of the genetic circuitry underpinning normal ageing may lead to a better understanding of, and therefore opportunities to intervene in, age-related disease, an increasing burden on society in an ageing population.

As an organism ages, there is no overt change in the genome sequence, other than the accumulation of somatic mutations which may give rise to spontaneous tumours. However, it is becoming increasingly clear that ageing organisms undergo profound changes in the gene expression patterns, resulting from degeneration of their chromatin (reviewed in [6]). The heterochromatin loss model of ageing was first introduced in the late 1990s, theorising that global heterochromatin loss could drive changes in cellular processes associated with ageing [7]. The heterochromatin island hypothesis emerged shortly after, proposing that the decreasing capacity of heterochromatin islands to reassemble after cell replication leads to transcriptional changes that promote ageing [8].

There is considerable experimental evidence supporting the idea that repressed epigenetic states stabilising developmental or reproductive gene expression patterns (or preventing genome instability) are gradually lost as organisms age. For example, cells isolated from old individuals, or from patients suffering from premature ageing disorders, display a reduction in the level of chromatin marks associated with the repressed state [9, 10]. In addition, ageing is also correlated with a reduction in the global level of histone proteins in several organisms, which would also be expected to allow inappropriate access to DNA, thus deregulating the gene expression patterns [11–13]. But do such changes in chromatin composition drive the ageing process, or are they simply correlated with it?

The activity state of chromatin is governed by a whole host of chromatin-modifying enzymes that target core histones by acetylation, phosphorylation, ubiquitylation and methylation [6]. Although the relationship between histone modifications and chromatin activity states is complex, it varies according to the genomic context. Active chromatin is generally characterised by lysine acetylation and trimethylated lysine 4 on histone H3 (H3K4me3) while inactive chromatin has deacetylated lysines and trimethylated lysine 27 on histone H3 (H3K27me3) or lysine 9 (H3K9me3). Thus, interfering with the function of chromatin-modifying enzymes such as lysine methylases/demethylases and acetylases/deacetylases in tractable model organisms is a useful way of assessing whether changes in chromatin activity states can drive altered longevity outcomes.

Previous studies in *C. elegans* have implicated the H3K27 demethylase UTX-1 as being an important regulator of lifespan, with loss of function mutants (containing higher levels of H3K27me3) displaying increased lifespan [14, 15]. This correlates with data suggesting global loss of H3K27me3 in normally aged *C. elegans* and prematurely aged cells from Hutchinson-Guildford progeroid syndrome (HGPS) patients [14, 16, 17], thus supporting the heterochromatin loss model of ageing. However, the data are complex; other studies report mutation of the H3K27 methyltransferase E(Z) in *Drosophila* decreasing H3K27me3 levels but extending lifespan [18]. An additional complexity arises from the observation that UTX-1, at least with respect to its function during *C. elegans* development, does not require the catalytic demethylase domain [19], raising the possibility that genes annotated as encoding a particular chromatin-modifying activity may not in fact utilise this activity for their in vivo functions.

With respect to H3K4 methylation marks normally associated with active chromatin, knockdown of the *C. elegans* H3K4 methyltransferase SET-2 (together with other members of the COMPASS complex ASH-2 and WDR-5) is associated with lower levels of H3K4me3 and extended longevity. Loss of the H3K4me3 demethylase RBR-2, on the other hand, shortens lifespan (while overexpression extends lifespan), consistent with the heterochromatin loss model of ageing. However, mutation of the H3K4 demethylase LSD-1 extends longevity [14, 15, 20]. Thus, chromatin modifiers associated with both active and inactive chromatin states appear to regulate lifespan, complicating the emerging picture that ageing is associated primarily with the loss of repressive chromatin. Possible explanations for the emerging complexity include tissue and target specific effects, as well as the potential for enzymes that modify the methylation state of lysine residues to have non-histone targets.

In order to take an unbiased approach to assess the role of chromatin regulators in regulating longevity in *C. elegans*, we performed an RNAi screen using a library of 330 highly conserved chromatin-related factors, identifying several genes which when knocked down by RNAi caused lifespan extension. We found both lysine methyltransferase and demethylases associated with enhanced longevity, all of which have been suggested to act at H3K27. Strikingly, overexpression, as well as loss-of-function, of two putative demethylases acting on H3K27, *jmjd-3.2* and *utx-1*, both caused lifespan extension. Furthermore, lifespan extension by JMJD-3.2, but not UTX-1 overexpression, was found to be independent of the demethylase domain, contrasting with the function of UTX-1 in development, which does not require the demethylase domain [19]. Finally, we investigated whether RNAi or overexpression of *utx-1* in specific tissues causes lifespan extension, finding distinct tissue-specific effects.

## Results

### Chromatinome RNAi screen for longevity regulators

We set up a blind RNAi screen in which worms were inspected for the altered accumulation of lipofuscin (an age-related autofluorescent pigment) at day 3 and day 6 of adulthood after continuous exposure to RNAi-feeding bacteria from the parental generation. Worms were scored by eye under a fluorescence microscope for significantly increased or decreased accumulation of lipofuscin compared to controls (see Additional file 1: Fig. S1 for examples). RNAi clones producing clear differences in lipofuscin accumulation when fed to worms were then tested in a secondary screen involving full lifespan analysis. Clones that resulted in significant lifespan extension upon RNAi knockdown included *mes-2*, *jmjd-3.2*, *cbp-1* and *isw-1* (Additional file 2: Table S1).

These four genes are representative of distinct families of chromatin regulating factors. *isw-1* (yeast homologue *ISW2*, human homologue Smrca1) encodes the catalytic subunit of the ATP-dependent nucleosome remodelling enzymes (NURF complex) having rather general roles in chromatin remodelling [21]. *cbp-1* is the *C. elegans* homologue of p300/CBP transcription cofactors suggested to have lysine acetyltransferase (KAT) activity [22, 23]. The other two factors identified in the screen, *mes-2* and *jmjd-3.2*, encode a lysine methyltransferase and demethylase, respectively, with shared substrates (H3K27 being the most widely reported), and we chose to focus our subsequent analysis on these two classes of enzymes because of their related activities. *mes-2* (which has been previously isolated in an RNAi-based longevity screen [18]) encodes the single *C. elegans* SET domain-containing lysine methyltransferase, known to associate with MES-3 and MES-6 to form the *C. elegans* PRC2 complex. RNAi of *mes-3* and *mes-6*, similarly to *mes-2*, gave rise to lifespan extension (Additional file 2: Table S1).

**Fig. 1:**
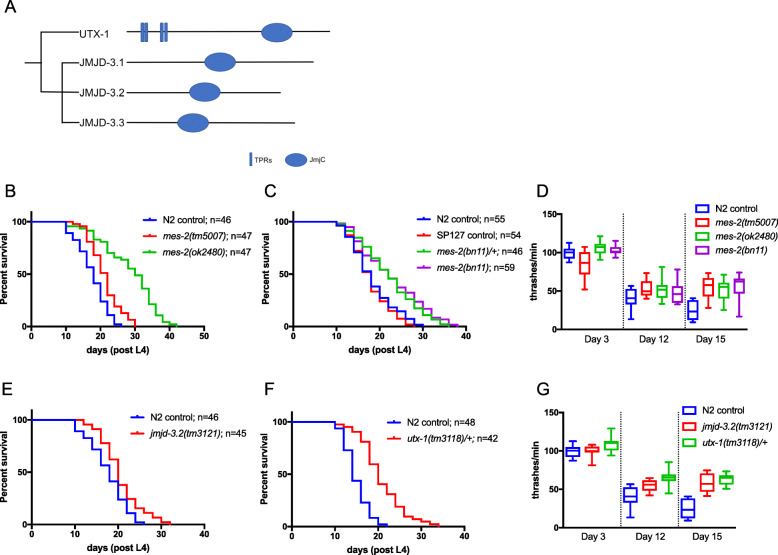
Lifespan and healthspan regulation by H3K27 methylases and demethylases. **a** The KDM6 family of H3K27 demethylases comprised *utx-1*, *jmjd-3.1*, *jmjd-3.2* and *jmjd-3.3* in *C. elegans* (TPR, tetratricopeptide repeat; JmjC, Jumonji C domain). Animals containing mutant alleles of *mes-2*, *jmjd-3.2* or *utx-1* were analysed for both lifespan (**b**, **c**, **e**, **f**) and healthspan (**d**, **g**). **b** Both *mes-2(tm5007)* and *mes-2(ok2480)* mutants live significantly longer than the N2 control strain (***p = 0.0003 and ****p < 0.0001, respectively). **c** Worms that are either homozygous or heterozygous for the *mes-2(bn11)* allele have extended lifespan when compared to either N2 (****p < 0.0001 and ***p = 0.0005, respectively) or the balancer-containing (SP127) control (****p < 0.0001). **d** The three *mes-2* mutants display an increased thrashing rate, indicative of better health, at day 12 and day 15 of adulthood compared to the N2 control strain (*p* < 0.005 in all cases). **e**, **f**: *jmjd-3.2(tm3121)* mutant worms (**e**) and *utx-1(tm3118)* heterozygotes (**f**) display extended lifespan compared to the N2 control (**p = 0.005 and ****p < 0.0001, respectively). **g** *jmjd-3.2(tm3121)* mutants and *utx-1(tm3118)/+* heterozygotes display increased thrashing rates, indicating better health, at day 12 and day 15 of adulthood when compared to N2 controls (*p* < 0.001 in each case) (see Additional file 3: Table S2 for the full statistical analysis of lifespan data, including repeats). For thrashing assays, *n* ≥ 10 worms, measured at least 3 times for each strain. Box and whisker plot represents the first and third quartile and minima and maxima of the data points

*jmjd-3.2* forms part of the larger KDM6A family of lysine demethylases (Fig. 1a), which includes two paralogs, *jmjd-3.1* and *jmjd-3.3*, as well as *utx-1*. The close homology of JMJD-3.2 to mammalian JMJD3 suggests a putative demethylase function against H3K27me3. Indeed, western blot experiments have shown that H3K27me3 levels are elevated (and H3K27me1/2 levels concomitantly reduced) in *jmjd-3.1;jmjd-3.2;jmjd-3.3* triple mutants compared to wild-type worms [19]. We tested the three other family members by RNAi, finding that *utx-1* (as previously reported by [14, 15]), but not *jmjd-3.1* or *jmjd-3.3* knockdown, caused lifespan extension (Additional file 2: Table S1).

### Mutations in H3K27 methyltransferases and demethylases prolong lifespan and healthspan

We confirmed the RNAi results by investigating lifespan in *mes-*2, *jmjd-3.2* and *utx-1* mutant alleles. The three *mes-2* alleles tested all had significantly extended lifespan (Fig. 1b, c; Additional file 3: Table S2). *mes-2(ok2480)* and *mes-2(bn11)* (both likely null alleles) had the strongest lifespan-enhancing effect while *mes-2(tm5007)* (a likely hypomorph) had a more modest effect. Furthermore, heterozygotes for the strong loss of function *mes-2*(*bn11*) allele also displayed lifespan extension (Fig. 1c; Additional file 3: Table S2), indicating the dominance of the longevity phenotype in this mutant.

To investigate whether the extended lifespan of *mes-2* mutants is associated with improved healthspan (i.e. better health in old age), we assessed the age-related movement deficit in a liquid thrashing assay in which wild-type worms display a marked motility deficit by day 12 of adulthood. All three *mes-2* alleles reduced the decline in the age-related movement to a similar extent (Fig. 1d), suggesting that only partial loss of *mes-2* function, such as in the *mes-2(tm5007)* allele, is sufficient to improve the healthspan of worms.

Next, we tested *jmjd-3.2* and *utx-1* mutants, finding similar improvements in both lifespan and healthspan (Fig. 1e–g; Additional file 3: Table S2). In the case of *utx-1* mutants, which have various developmental defects and do not survive to adulthood, heterozygotes were used (Fig. 1f, g; Additional file 3: Table S2), again suggesting that lifespan and healthspan extension is a dominant phenotype in *utx-1* mutants, similar to *mes-2*. Consistent with the lack of lifespan extension as a result of RNAi knockdown of *jmjd-3.1* and *jmjd-3.3* (Additional file 2: Table S1), the *jmjd-3.1; jmjd-3.2; jmjd-3.3* triple mutant did not display any increase in lifespan over the *jmjd-3.2* single mutant (Additional file 4: Fig. S2; Additional file 5: Table S3).

### Enhanced longevity of *mes-2*, *utx-1* or *jmjd-3.2* mutants is independent of reproductive status

One possible explanation for the increased lifespan of certain strains is the reduced developmental rate. For example, several mitochondrial mutants develop very slowly, and their prolonged longevity can be attributed to this reduced “rate of living” [24, 25]. In the case of *mes-*2, *jmjd-3.2* and *utx-1*(+/−) mutants, however, the developmental rate was comparable with wild-type animals (Additional file 6: Fig. S3).

Fertility is also known to be tightly linked to lifespan, with sterile animals often displaying longer lifespans, as changes in the allocation of energy resources between tissues in animals with a malfunctioning germline are thought to promote longevity [26]. However, not all sterile animals live longer, and not all longer-lived animals are sterile. We assessed the fertility of mutant strains by measuring the brood size (Table 1). No change in the brood size of *utx-1* (heterozygotes) or *jmjd-3.2* mutants was observed, despite the lifespan extension phenotype. As expected, a reduction in brood size was observed in *mes-2* mutants (Mes stands for maternal effect sterile), although this was allele-specific, with F2 *mes-2(ok2480)* and *mes-2(bn11)* homozygotes being completely sterile (Table 1). *mes-2(tm5007)* mutants had a normal brood size, and *mes-2(bn11)/+* heterozygotes displayed only a modest reduction in the brood size, despite the fact that both these strains were long-lived (see Fig. 1; Additional file 3: Table S2). Taken together, these results suggest that regulation of lifespan by these epigenetic modifiers acts independently of reproduction.

**Table 1:** Reduced brood size of *mes-2*, *utx-1* and *jmjd-3.2* mutants is not required for lifespan extension. Single worms were picked onto separate plates and the whole brood counted by moving the mother onto a fresh plate each day until egg-laying stopped and combining the counts for each plate. The broods of a minimum of 10 animals were assessed per strain. The table is ranked by brood size. *p* values are relative to the wild-type N2 strain

Genotype	Average brood size ± SEM	*p* value	Significance
N2	238 ± 7	–	–
*mes-2(tm5007)*	231 ± 7	> 0.999	ns
*utx-1(tm3118)/+*	230 ± 8	0.999	ns
*jmjd-3.2(tm3121)*	228 ± 14	0.989	ns
*mes-2(bn11)/+*	176 ± 7	< 0.001	***
*mes-2(bn11)*	0	< 0.001	***
*mes-2(ok2480)*	0	< 0.001	***

### *daf-16* dependence of *mes-2*, *jmjd-3.2* and *utx-1* associated lifespan extension

**Fig. 2:**
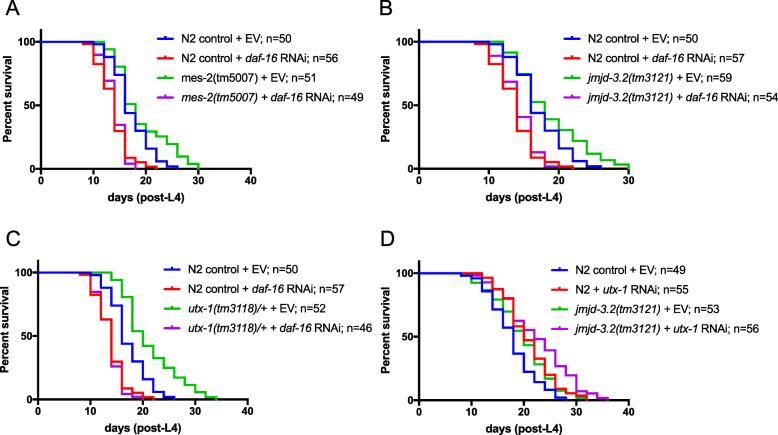
Pathway analysis of *mes-2*, *jmjd-3.2* and *utx-1* mediated lifespan extension. *mes-2*, *jmjd-3.2* and *utx-1* mutant alleles were subjected to *daf-16* RNAi and lifespan assays performed in order to investigate possible lifespan-extending pathways. **a**–**c** RNAi depletion of *daf-16* in *mes-2(tm5007)* (**a**), *jmjd-3.2(tm3121)* (**b**) and *utx-1(tm3118)/+* (**c**) backgrounds completely suppresses lifespan extension. **d** Combinatorial knockdown of *jmjd-3.2* (using mutant allele *tm3121)* and *utx-1* (using post-embryonic RNAi) results in increased life extension compared to either the *jmjd-3.2* allele (**p = 0.007) or *utx-1* RNAi alone (*p = 0.03). Controls are shared between experiments **a**, **b** and **c** (as the experiments were performed as a large set) although the graphs are separated for clarity. EV, empty vector control (i.e. worms fed HT115 bacteria transformed with L4440 RNAi vector lacking a genomic insert) (see Additional file 7: Table S4 for the full statistical analysis of data, including repeats)

The striking result from the RNAi screen and subsequent analysis of mutants was that reduced levels of both methyltransferase and demethylases known to target H3K27 in chromatin extend both lifespan and healthspan. Do these enzymes have different targets, perhaps working in different pathways? The insulin/IGF signalling (IIS) pathway is known to be central to the regulation of lifespan in several organisms, and a convenient way to assess whether lifespan-extending mutations are associated with perturbations in IIS is to knock down *daf-16*, the FOXO transcription factor central to the pathway [27, 28]. RNAi of *daf-16* completely suppressed the lifespan extension of all three mutants, *mes-2*, *jmjd-3.2* and *utx-1* (Fig. 2a–c; Additional file 7: Table S4), suggesting that all three genes control longevity in a *daf-16*-dependent manner. In the case of *utx-1*, this confirms a previous report [14, 15]. Given that *utx-1* and *jmjd-3.2* are both H3K27 demethylases, we tested for redundancy by subjecting *jmjd-3.2* mutants to *utx-1* RNAi. We found that *utx-1* RNAi further increased the lifespan of *jmjd-3.2* mutants (Fig. 2d; Additional file 7: Table S4). This suggests either partial redundancy or that the two genes act in separate lifespan regulatory pathways that converge on *daf-16*.

One of the mechanisms by which DAF-16 is known to regulate longevity is via translocation to the nucleus in long-lived animals [27, 29]. To investigate DAF-16 localisation when the expression of *mes-2, jmjd-3.2* or *utx-1* was reduced, we performed RNAi experiments in strain TJ356, containing an integrated DAF-16::GFP translational reporter [14]. We found that nuclear DAF-16 was increased when either *mes-2* or *utx-1* were knocked down by RNAi, with *jmjd-3.2* having a more marginal effect (Additional file 8: Fig. S4). Taken together, these data are consistent with these genes acting through the IIS pathway to regulate longevity, although it is possible that *jmjd-3.2* impinges on DAF-16 by some means other than causing nuclear translocation.

### Overexpression of *jmd-3.2* and *utx-1* results in lifespan extension

**Fig. 3:**
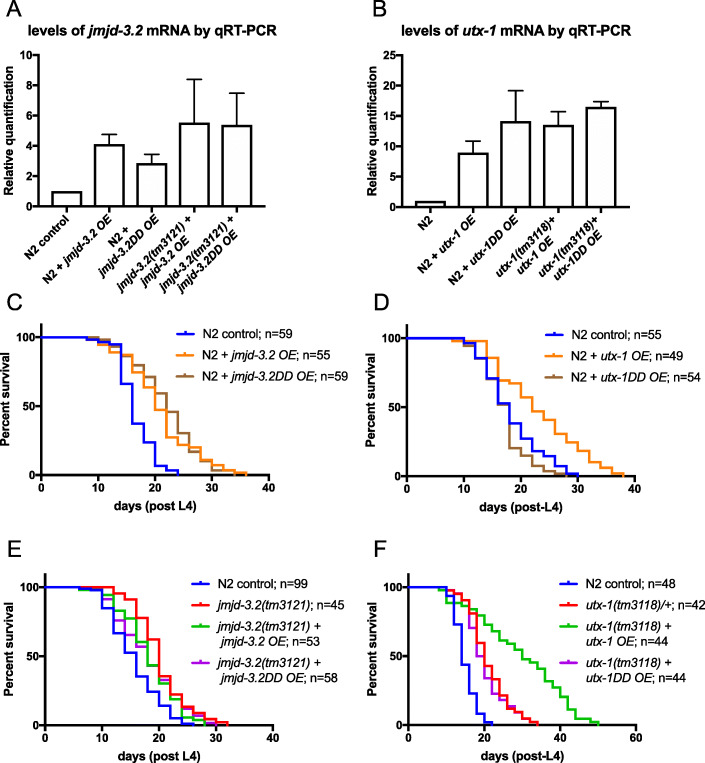
Overexpression of *jmjd-3.2* or *utx-1* causes lifespan extension. **a**, **b** Levels of *jmjd-3.2* (**a**) and *utx-1* mRNA (**b**) were assessed by qRT-PCR, showing significant upregulation in the *jmjd-3.2* and *utx-1* transgenic lines, respectively, when either wild-type or demethylase dead (DD) constructs were used. Error bars represent the SEM for each data point. Lifespan assays were performed on transgenic lines overexpressing *jmjd-3.2* or *utx-1* driven by their respective endogenous promoter. **c**, **d** overexpression of either *jmjd-3.2* (**c**) or *utx-1* (**d**) in a wild-type background results in lifespan extension (****p < 0.0001). Overexpression of demethylase dead *jmjd-3.2* (*jmjd-3.2DD*) also results in lifespan extension (****p < 0.0001), whereas overexpression of demethylase dead *utx-1* (*utx-1DD*) has no effect. **e** Overexpression of either wild-type *jmjd-3.2* or demethylase dead *jmjd-3.2* (*jmjd-3.2DD*) does not further extend the lifespan of *jmjd-3.2(tm3121)* mutants. **f** Overexpression of wild-type *utx-1* in a *utx-1*(*tm311*8) mutant background promotes lifespan extension beyond that of the long-lived *utx-1*(*tm3118/+*) mutant strain (****p < 0.0001), whereas overexpressing the demethylase dead version has no effect (see Additional file 9: Table S5 for full statistical analysis of lifespan data, including repeats). (OE, overexpression; DD, demethylase dead). N2 control strains contained the same coinjection marker (*rol-6*^*+*^) as the *jmjd-3.2* and *utx-1* transgenic lines

Intrigued by the observation that depletion of enzymes of predicted opposing functions (H3K27 methyltransferase and demethylases) both resulted in extended lifespan, we next investigated the effect of *jmjd-3.2* and *utx-1* overexpression on worm longevity. We used transgenic worms carrying full-length gene constructs of *jmjd-3.2* and *utx-1* driven by their respective endogenous promoters as multicopy extrachromosomal arrays in a wild-type (WT) background. In both cases, overexpression (as monitored by qRT-PCR, Fig. 3a, b) resulted in significant lifespan extension (Fig. 3c, d; Additional file 9: Table S5). Intriguingly, the demethylase activity of UTX-1 has been shown to be dispensable for worm development [19]. We therefore tested whether UTX-1 and JMJD-3.2 demethylase domains were necessary for lifespan extension in the context of overexpression by constructing transgenic strains expressing “demethylase dead” (DD) versions of each enzyme and measuring lifespan. In the case of *jmjd-3.2*, mutation of the putative demethylase domain (based on protein sequence homology) had no effect on its ability to extend lifespan in a WT background (Fig. 3c; Additional file 9: Table S5), suggesting that lysine demethylase activity is not required for regulation of lifespan by *jmjd-3.2* overexpression. In contrast, mutation of the UTX-1 demethylase domain completely suppressed lifespan extension upon overexpression in the WT background (Fig. 3d; Additional file 9: Table S5), indicating that the *utx-1* demethylase domain is required for lifespan extension in this context, contrary to its effects during development.

We also investigated the longevity outcome when these overexpressing transgenes (the same extrachromosomal array in each case) were crossed into *jmjd-3.2* and *utx-1* mutant backgrounds. Lifespan extension over the N2 control was observed in all cases (Fig. 3e, f; Additional file 9: Table S5). It is noteworthy that overexpression of *utx-1* in a *utx-1(tm3118)* mutant background resulted in a much more marked lifespan extension than either overexpression in the wild-type background or in the *utx-1(tm3118)* mutant background alone. The comparison can be made by comparing the data in Fig. 3d with 3F. Overexpressing *utx-1* in a wild-type background gave a 26% increase in mean lifespan over the control, whereas overexpressing in a *utx-1* mutant background gave a much bigger increase (99%). However, in comparing these values, it is necessary to take into account the effect of the mutant background itself on lifespan. Lifespan in *utx-1* mutants is already 41% higher than the N2 control in this experiment, but overexpressing *utx-1* in this background leads to a further 41% increase, i.e. the effects of *utx-1* loss and gain of function are additive. It seems unlikely that this is solely to do with expression levels, given that both loss and gain of *utx-1* expression results in lifespan extension. Rather, this suggests that raising or lowering the level of *utx-1* might impinge on alternative longevity pathways. Overexpression of *jmjd-3.2* in a *jmjd-3.2(tm3121)* background, on the other hand, did not increase lifespan over and above that of the *jmjd-3.2* mutant alone or *jmjd-3.2* overexpression in a wild-type background, i.e. loss and gain of *jmjd-3.2* function have similar effects.

Consistent with the data discussed above, mutation of the *jmjd-3.2* demethylase domain had no effect on its ability to extend lifespan in a *jmjd-3.2(tm3121)* background (Fig. 3e; Additional file 9: Table S5), whereas expression of the mutated *utx-1* prevented the extra lifespan extension observed in *utx-1(tm3118)* mutant animals overexpressing wild-type *utx-1* (Fig. 3f; Additional file 9: Table S5), again suggesting that demethylase activity is absolutely required for lifespan extension mediated by *utx-1*, but not *jmjd-3.2*, overexpression. Given the lack of dependence of *jmjd-3.2* mediated lifespan regulation on its putative demethylase activity, we focussed on *utx-1* only for our subsequent analysis.

### Elevated *utx-1* expression makes worms more resistant to environmental stressors

**Fig. 4:**
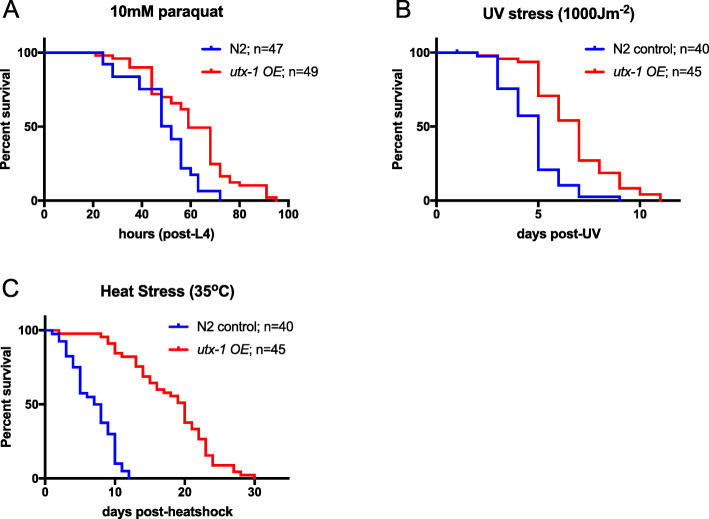
Overexpression of *utx-1* enhances resistance to oxidative, UV and heat stressors. Animals were either exposed to 10 mM paraquat from L4 onwards to induce oxidative stress (**a**), given a 1000-Jm^−2^ dose of UVC 3 days post-L4 to induce UV stress (**b**), or exposed to 35 °C heat for 6 h from 5 days post-L4 to induce heat stress (**c**), and scored for survival. In all cases, animals overexpressing *utx-1* (red survival curve) were more resistant to stress compared to control worms containing the same co-injection marker (*rol-6*^*+*^) (blue survival curve) (all ****p < 0.0001) (see Additional file 10: Table S6 for the full statistical analysis of lifespan data, including repeats)

We were intrigued to investigate whether overexpression of *utx-1* increases worm health, as well as lifespan, similar to what we observed for *utx-1* mutants (Fig. 1). We could not test age-related movement decline in the overexpressors, though, because the transgenic strains we constructed contained the *rol-6* marker that affects worm movement, confounding results from thrashing assays. Instead, we investigated tolerance to oxidative, UV and heat stress. In all three cases, overexpression of *utx-1* led to enhanced survival in stressful conditions (Fig. 4a–c; Additional file 10: Table S6). Increased tolerance of oxidative, UV and heat stress has been previously reported in *utx-1(RNAi)* animals [14]. Taken together, this suggests that *utx-1* overexpression, similar to *utx-1* loss of function, increases overall health as well as lifespan.

### Interaction of *utx-1* with insulin signalling in lifespan regulation

**Fig. 5:**
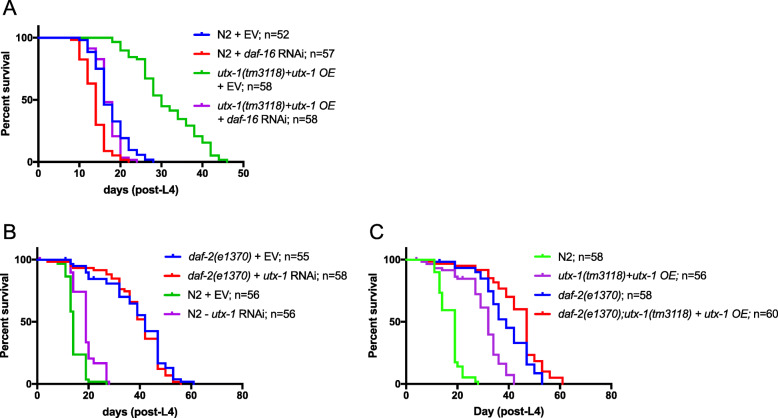
Lifespan extension due to *utx-1* overexpression is independent of insulin signalling but at least partially dependent on *daf-16*. **a** Lifespan assays were performed on transgenic animals overexpressing *utx-1* in a *utx-1(tm3118)* mutant background combined with *daf-16* RNAi. *daf-16* depletion significantly shortened the lifespan of wild-type and transgenic animals compared to EV controls (****p < 0.0001), although *utx-1* overexpressing animals subjected to *daf-16* RNAi did display a moderate lifespan extension compared to wild-type *daf-16*(RNAi) animals (****p < 0.0001). **b** Lifespan assays performed on *daf-2(e1370)* mutants subjected to *utx-1* RNAi resulted in no significant change in survival compared with *daf-2(e1370)* EV controls, consistent with findings from [14]. The lifespan increase upon *utx-1* RNAi in a wild-type background confirms that the *utx-1* RNAi was working effectively. **c** Lifespan assays performed on *daf-2(e1370)* mutants compared to *daf-2(e1370)* mutants overexpressing *utx-1* showed a significant lifespan extension compared with *daf-2(e1370)* mutants alone (**p = 0.0023) or *utx-1* overexpression alone. EV, empty vector control (i.e. worms fed HT115 bacteria transformed with L4440 RNAi vector lacking a genomic insert); OE, overexpression (see Additional file 11: Table S7 for the full statistical analysis of lifespan data, including repeats)

*utx-1* loss of function has been previously reported to increase H3K27 methylation of the *daf-2* promoter [14], consistent with the observed *daf-16* and *daf-2* dependence of lifespan extension due to *utx-1* RNAi (Fig. 2c and [14]). This provokes the question of how elevated expression of *utx-1* might impinge on insulin signalling to promote longevity. To investigate this, we first depleted *daf-16* by RNAi in *utx-1* overexpressing animals. Intriguingly, we found that lifespan extension due to *utx-1* overexpression is only partially *daf-16* dependent, as overexpression of *utx-1* significantly increases the lifespan of *daf-16* RNAi animals (Fig. 5a; Additional file 11: Table S7). DAF-16 is a known point of convergence in several different cellular signalling pathways, including but not limited to the insulin/IGF1 pathway. Therefore, to clarify the specific role of insulin/IGF1 signalling in *utx-1* regulated lifespan, we tested both loss and gain of *utx-1* function in a *daf-2* mutant background. Consistent with the previous report [14], we found that *utx-1* RNAi does not further increase the lifespan of *daf-2* mutants (Fig. 5b; Additional file 11: Table S7). However, when we overexpressed *utx-1* in *daf-2* mutants, a significant increase in lifespan was observed, over and above that of the *daf-2* mutants alone (or *utx-1* overexpression alone) (Fig. 5c; Additional file 11: Table S7). This suggests that the mechanism governing *utx-1* overexpression-mediated longevity is, at least in part, independent of the insulin/IGF1 signalling pathway.

### *utx-1* regulates lifespan in a tissue-specific manner

*utx-1* and *jmjd-3.2* overexpression appear to influence lifespan by distinct mechanisms (with the demethylase domain appearing to be dispensable in the case of *jmjd-3.2*), possibly involving different molecular targets or expression in different tissues. Consistent with the latter hypothesis, we observed that the two demethylases have distinct expression patterns; expression of *jmjd-3.2::gfp* under the endogenous promoter appears to be restricted to a subset of neurons, while *utx-1::gfp* expression is ubiquitous (Additional file 12: Fig. S5).

**Fig. 6:**
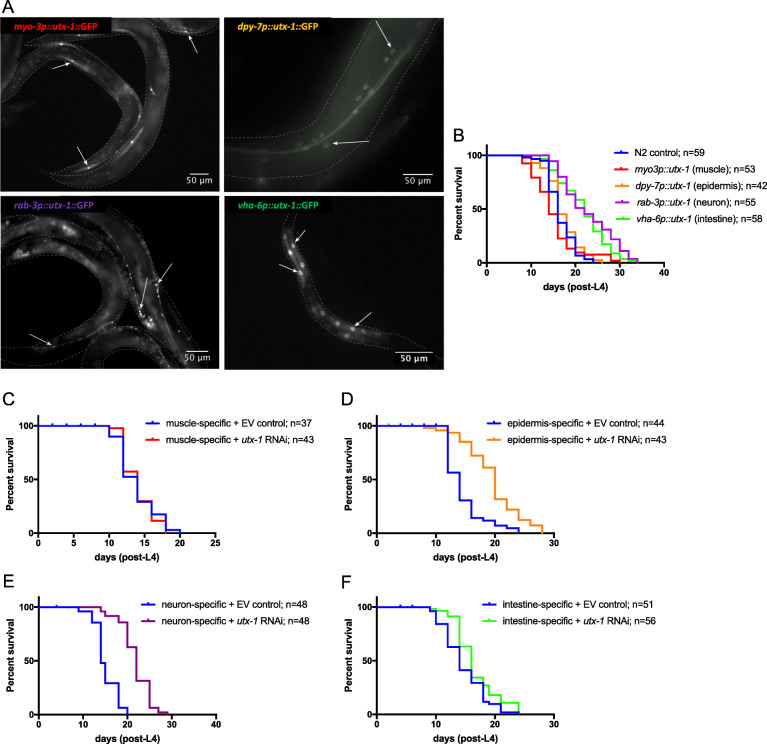
Gain or loss of *utx-1* expression in specific tissues induces lifespan extension. **a** Expression of GFP in transgenic lines was monitored to confirm tissue-specific expression. Top left panel: muscle cell expression of *utx-1::gfp* driven by the *myo-3* promoter (white arrows); top right panel: epidermal expression driven by the *dpy-7* promoter (white arrows); bottom left panel: neuronal expression driven by the *rab-3* promoter (white arrows); bottom right panel: intestinal expression driven by the *vha-6* promoter (white arrows). White dashed line is the outline of the worm in each case. **b** Lifespan assays were performed on transgenic animals overexpressing *utx-1* in specific tissues. Lifespan extension was observed when *utx-1* was overexpressed in neuronal and intestinal cells (****p < 0.0001 in both cases), but not in epidermal or muscle cells. **c**–**f** Lifespan assays were performed in animals subjected to tissue-specific knockdown of *utx-1* by RNAi. In the case of muscle, epidermal and intestinal knockdown, the RNAi insensitive *rde-1(ne219)* mutant was used, rescued by muscle- (**c**), epidermal- (**d**) or intestinal-driven (**f**) *rde-1*, and animals subjected to *utx-1* RNAi post-embryonically. For neuronal knockdown (**e**), *sid-1* was expressed pan-neuronally in the RNAi-insensitive *sid-1* mutant background, and *utx-1* RNAi performed post-embryonically. Marked lifespan extension was seen in epidermal and neuronal knockdown (****p < 0.0001 in each case), slight lifespan extension was seen in intestinal knockdown (**p = 0.009) and no lifespan extension was associated with muscle-specific knockdown. EV, empty vector control (i.e. worms fed HT115 bacteria transformed with L4440 RNAi vector lacking a genomic insert) (see Additional file 13: Table S8 for the full statistical analysis of lifespan data, including repeats)

Given that *jmjd-3.2* is expressed only in neurons, the lifespan-extending effect of both elevated and reduced *jmjd-3.2* expression is presumably neuron-specific (although different subsets of neurons could be involved). However, *utx-1* is ubiquitously expressed; therefore, it was of interest to investigate the tissue-specific basis of lifespan extension caused by either gain or loss of *utx-1* function. To analyse this, we used transgenic worms expressing full-length GFP-tagged *utx-1* driven by tissue-specific promoters, confirming the correct expression (Fig. 6a) before measuring their lifespan. We found that overexpression of *utx-1* in the epidermis or muscle did not cause lifespan extension, whereas overexpression in either neurons or intestine both caused lifespan extension to similar extents (Fig. 6b; Additional file 13: Table S8). The *daf-16* dependence of lifespan extension due to neuronal and intestinal overexpression of *utx-1* was tested by subjecting the animals to *daf-16* RNAi. In both tissue-specific overexpression strains, the lifespan of *daf-16*-depleted animals was significantly shortened compared to tissue-specific overexpression EV controls. However, the longevity of these animals was still improved compared to wild-type worms subjected to *daf-16* RNAi, suggesting partial *daf-16* independence (Additional file 14: Fig. S6A and B; Additional file 15: Table S9).

Next, we performed tissue-specific RNAi knockdown of *utx-1*. In the case of muscle, intestine- and epidermal-specific RNAi, we took advantage of an *rde-1* RNAi-insensitive mutant rescued by tissue-specific expression of *rde-1* cDNA, thus rendering specific tissues RNAi sensitive [30]. To achieve neuron-specific knockdown we used the RNAi insensitive *sid-1* strain rescued by neuron-specific expression of SID-1 [31]. Using these strains, we found that *utx-1* RNAi in the epidermis, intestine and neurons results in lifespan extension, while loss of *utx-1* in muscle has no effect (Fig. 6c–f; Additional file 13: Table S8). It is noteworthy that the tissues sensitive to lifespan extension due to *utx-1* knockdown (epidermis, neurons and intestine) are exactly the same as the tissues involved in *daf-2* mediated lifespan regulation [32] (Additional file 16: Fig. S7; Additional file 17: Table S10). The *daf-16* dependence of these lifespan increases due to tissue-specific knockdown of *utx-1* was determined by repeating the lifespan analysis in a *daf-16* mutant background (Additional file 14: Fig. S6C-F; Additional file 15: Table S9). We found no statistical difference between lifespans associated with neuronal and intestinal specific *utx-1* RNAi in a *daf-16(mu86)* mutant background and the *daf-16* empty vector controls, suggesting that enhanced longevity observed when *utx-1* was knocked down in these tissues was completely dependent on *daf-16*. Lifespan associated with epidermis-specific *utx-1* RNAi in a *daf-16(mu86)* mutant background was slightly longer than the *daf-16* control, but the effect was marginal; therefore, it is difficult to make firm conclusions about *daf-16* dependency of lifespan extension mediated by epidermis-specific *utx-1* RNAi. Overall, therefore, we conclude that lifespan extension caused by *utx-1*overexpression (either ubiquitously or in specific tissues) displays some degree of *daf-16* independence, whereas *utx-1* depletion causes *daf-16*-dependent lifespan extension.

## Discussion

Using a non-biassed, lipofuscin-based pre-screen for longevity regulators followed by full lifespan analysis of potential hits, we identified four chromatin-regulating genes that extend lifespan in *C. elegans*: *mes-2*, *cbp-1*, *isw-1* and *jmjd-3.2*. This group includes members of two antagonistic classes of H3K27 methylation modifiers, the methyl transferase *mes-2* and demethylase *jmjd-3.*2. *mes-2* has been previously identified in an RNAi screen for longevity genes, giving a modest 7.5% increase in mean lifespan [18] (we observed a 13% average increase in *mes-2(RNAi)* animals). Our identification of *jmjd-3.2* as a longevity gene prompted us to test the other members of the KDM6A family of H3K27 demethylases, *utx-1*, *jmjd-3.1* and *jmjd-3.*3. We found, in agreement with previous reports [14, 15], that loss of *utx-1* function extends lifespan. These previous studies have reported average increases in mean lifespan of 25% [14] and 19% [15] in *utx-1(RNAi)* animals, and consistent with this, we observed an average increase of 26%. Previous studies of *utx-1(tm3118/+)* animals have reported 13% [14] and 39% [15] average increases in mean lifespan, compared with our 30%. In contrast, we found no lifespan regulatory role for either *jmjd-3.1* or *jmjd-3.3.* The involvement of *jmjd-3.2* in lifespan regulation has not been reported before, possibly due to the logistical and methodological differences in the way this screen and previous ones were conducted. Such differences could account for the variation between our screen’s identification rate (4/330 = 1.2%) and those reported in other longevity screens [33–36]. Overall, our findings corroborate the results of several of these screens, further highlighting the remarkable longevity-regulating capacities of lysine methyltransferases and demethylases.

Our analysis of mutant phenotypes confirmed the RNAi results, generally giving more robust effects. Strikingly, heterozygous mutants of *mes-2* and *utx-1* displayed robust lifespan extension. The dominance characterising the lifespan phenotype suggests haploinsufficiency of these genes for lifespan regulation, a feature of genes whose protein products are required at high concentration. It is intriguing that heterozygous *utx-1* mutants are long-lived, suggesting the dominance of *utx-1* mutants for the extended lifespan phenotype, whereas developmental phenotypes associated with loss of *utx-1* function are completely recessive [19]. This suggests that different levels of UTX-1 may be associated with different life stages, and is consistent with reports suggesting that levels of *utx-1* increase with age, in both humans and worms [14]. Furthermore, it is intriguing that *utx-1* heterozygotes display a lifespan extension that is at least comparable to, if not greater than, *utx-1(RNAi)* animals (see above), supporting the idea that lifespan regulation is very sensitive to *utx-1* dose. Likewise, *mes-2(bn11)* heterozygotes were also long-lived (displaying an average mean lifespan increase of 18%, compared with 13% in RNAi and 25% in homozygotes), suggesting at least partial dominance for lifespan, whereas *bn11/+* heterozygotes did not display the full sterility phenotype associated with homozygous mutants. Thus, similar to UTX-1, the requirements for MES-2 activity appear to be different for lifespan and fertility, suggesting that these two phenotypes are, at least in part, separable and potentially require different amounts of protein.

Importantly, long-lived animals produced by loss of *mes-2*, *jmjd-3.2* or *utx-1* function (and *utx-1* overexpression) were healthier in old age, as assayed by monitoring age-related movement deficit (or stress resistance in the case of *utx-1* overexpression). This suggests that H3K27 methyltransferases and demethylases are associated not just with lifespan, but also with healthspan.

Studies investigating the effects of H3K27 methyltransferases on lifespan have also been conducted in other model organisms, yielding similar results. For example, heterozygous mutation of PRC2 components E(Z) (homologue of MES-2) and ESC (homologue of MES-6) decreases global H3K27me3 levels in *Drosophila* yet extends lifespan [37], consistent with our findings in *C. elegans*. Additionally, we show that MES-3, a subunit of the worm PRC-2 complex with no apparent evolutionary relationship to any other Polycomb protein, also plays a role in longevity modulation. Analyses of recombinant PRC-2/MES sub-complexes in vitro have demonstrated that the assembly of an enzymatically active MES complex is contingent on the presence of all three of its constituent subunits [38]. Taken together, these findings point towards a model where the worm PRC-2/MES complex behaves like a holoenzyme to regulate lifespan, with MES-6 and MES-3 acting synergistically with MES-2 to potentiate its methyltransferase activity. Thus, depletion of either *mes-3* or *mes-6* would result in a loss of *mes-2* function and a concomitant increase in worm lifespan (also observed as a result of RNAi or mutation of *mes-2* itself). In contrast to the work on H3K27 methyltransferases, it has not been possible to directly test the lifespan of H3K27 demethylase mutants in flies, because the main homologue, UTX, is essential for survival.

The central question prompted by our results, is how both an elevation and a reduction of H3K27me3 levels (by knocking down both H3K27 demethylases and methyltransferase, respectively) promotes lifespan extension. According to the heterochromatin loss model of ageing, mutation of the demethylases (*utx-1, jmjd-3.2*) would be expected to increase lifespan, whereas loss of EZH2, the methyltransferase component of the PRC2 complex (*mes-2*), would be expected to reduce lifespan.

One possibility is that both classes of enzymes have non-histone substrates, although various reports support the importance of H3K27 as the dominant substrate in lifespan regulation. Firstly, H3K27me3 has been shown to decrease with age in worms [15], consistent with the heterochromatin loss model. Secondly, loss of PRC2 components is associated with a decrease in H3K27me3 [39, 40], whereas *utx-1* and *jmjd-3* depletion (in the triple *jmjd-3.1*, *jmjd-3.2*, *jmjd-3.3* triple mutant) leads to increased levels of H3K27me3 as shown by western blot [19]. In mammalian cells, UTX RNAi has been shown to increase the level of H3K27me3 but not to affect other marks (including H3K9me3, H3K4me3 and H3K36me3 [41]), again suggesting a high level of specificity. This notion is further supported by in vitro experiments showing that both human and *C. elegans* UTX-1 specifically demethylate H3K27me3 [14, 41].

Thus, increased lifespan is associated with what ought to be both lifespan-decreasing (lower levels of H3K27me3) and lifespan-increasing (higher levels of H3K27me3) chromatin signatures. The situation is made even more complex by our overexpression experiments, in which elevated expression of demethylase enzymes promotes longevity. Just as the loss of *utx-1* function has been previously linked to increased levels of H3K27me3, increased expression of *utx-1* can be directly linked to decreased levels of H3K27me3 in *C. elegans*, as shown by H3K27me3 antibody staining experiments. Indeed, we have previously shown that the decreased level of H3K27me3 observed in worms overexpressing *utx-1* was well correlated with the degree of overexpression (the more overexpression, the lower the level of H3K27me3) [19]. In contrast, overexpressing demethylase dead *utx-1* had no effect on H3K27me3 levels. Thus, we were able to conclude that *utx-1* functions as an H3K27 demethylase in vivo and that the catalytic domain is essential for this activity. Using the same transgenic strains, we have been able to show, in this report, lifespan extension upon *utx-1*, but not *utx-1DD* overexpression; therefore, it is appropriate for us to conclude that lifespan extension when *utx-1* is overexpressed is correlated with lower levels of H3K27me3.

In the case of *jmjd-3.2*, however, this appears to be rather different, as we find that the putative demethylase domain is dispensable for lifespan extension, at least with respect to overexpression, suggesting alternative mechanisms are at play. This is consistent with the fact that the mammalian homologue of *jmjd-3.2*, Jmjd3, has been shown to play a demethylase-independent role in chromatin remodelling to regulate T-box family member-dependent gene expression [42], although it is also the case that ectopic expression of JMJD3 in mammalian cells has been shown to decrease H3K27me3 levels [41]. The restricted expression pattern of *jmjd-3.2* in a small subset of neurons in *C. elegans* makes experiments to determine changes in H3K27me3 levels upon loss or gain of *jmjd-3.2* function very challenging in this organism, so it is not possible for us to make firm conclusions regarding the link between *jmjd-3.2* overexpression-induced lifespan extension and changes in H3K27me3 levels.

Given that H3K27me3 appears to be the dominant substrate for both loss and gain of *utx-1* function, it seems obvious to suggest that different levels of UTX-1 activity impinge on alternative molecular targets to drive alterations in lifespan, and this would explain why *utx-1* loss and gain of function are additive for lifespan extension. Previous reports have demonstrated increased H3K27me3 at the *daf-2* promoter when worms are subjected to *utx-1* RNAi, with the resultant reduction of *daf-2* expression conferring lifespan extension [14]. Thus, *utx-1* loss of function is not additive with *daf-2* loss of function ([14], confirmed in Fig. 5b). Moreover, we found that the tissues involved in lifespan extension due to *utx-1* knockdown (intestine, neurons, epidermis) are exactly the same as the tissues involved in *daf-2* mediated lifespan regulation, supporting the idea that *daf-2* is the principal target of UTX-1. However, we found that *utx-1* overexpression is additive with *daf-2*, suggesting the involvement of a different pathway. *daf-16* is involved in both cases (although we did note that *utx-1* overexpression induced lifespan extension does display a partial *daf-16* independence). *daf-2* and *daf-16* are interchangeably used as a readout for insulin signalling in lifespan analysis, but the example we present here urges caution in this respect, as DAF-16 is a known point of convergence in several cellular signalling pathways, not just IIS, and therefore, cellular processes can be *daf-16-*dependent yet *daf-2-*independent, or vice versa [43, 44].

Alternative molecular targets may therefore help to explain the pro-longevity effects of both loss and gain of function of *utx-1*. It has also been demonstrated that longevity regulation through perturbation of the IIS pathway might be partially attributed to the differential requirements of specific tissues for IIS components [32, 45, 46]. We found both overlap and differences in the tissue specificity of *utx-1* gain and loss of function. Both gain and loss of *utx-1* function in neurons and intestine promoted longevity. However, we found that lifespan extension caused by *utx-1* loss of function in both neurons and intestine was completely *daf-16* dependent, whereas gain of function associated longevity displayed some degree of *daf-16* independence. Thus, even in the same tissue, different levels of histone methylation can presumably impinge on alternative molecular targets to influence longevity outcomes. However, in the absence of direct tissue-specific H3K27 methylation measurements, any such interpretations are limited. In the epidermis, however, only loss of *utx-1* function caused lifespan extension. Epidermal cells are increasingly recognised as an important site of metabolic regulation [47]. Metabolism and ageing are intricately linked, suggesting that the epidermis is an overlooked tissue when it comes to ageing studies.

## Conclusions

Overall, our findings highlight the delicate context dependency characterising the lifespan regulatory roles of chromatin modifiers and suggest that extrapolations made on the basis of global histone methylation levels may be too generalised. Thus, the seemingly paradoxical association of a pro-longevity outcome with both loss and gain of function of both H3K27 methyltransferases as well as demethylases can be explained when different tissues and potentially distinct molecular targets are taken into account. While using whole organism, analysis is essential for understanding biological ageing, interpretation of the results can be challenging. For example, histone methylation levels have been routinely measured in whole worm extracts (thus averaging out potentially tissue-specific effects), and knockdown experiments are typically performed systemically. This may potentially occlude tissue-specific resolution, which could profoundly affect the interpretation of the mechanisms involved.

## Methods

### Genetics and strains

*C. elegans* strains were cultured using standard methods described in [48, 49]. The strains used are described in Additional file 18: Table S11.

*utx-1*(*tm3118)* is an out-of-frame deletion of 547 bp of *utx-1* resulting in early larval lethality in homozygous mutants [19]. *jmjd-3.2(tm3121)* is a complex substitution mutation deleting 356 bp (and inserting 13 bp), resulting in a frame shift.

*mes-2(bn11)* and *mes-2(ok2480)* alleles have been previously described as null mutations in the literature [39, 50]. *bn11* is a single nucleotide substitution resulting in a premature stop codon at the beginning of the second exon. *ok2480* is a complex substitution in which 1262 bp are deleted, and 3 bases are inserted leading to a frameshift. *mes-2(tm5007)* is a 164-bp deletion that causes loss of part of the promoter and beginning of the first exon.

### Brood size measurement

Single L4 stage larvae were placed on standard agar plates with OP50 bacteria at 20 °C and transferred to a new plate every 24 h. Viable progeny were counted every day until the F1 worm stopped laying eggs, and the counts for each plate were combined. The broods of a minimum of 10 animals were assessed per strain. The average number of progeny per worm is presented.

### RNA interference (RNAi)

The RNAi chromatinome library which is a re-grid of the Ahringer *C. elegans RNAi* feeding library [51] was kindly gifted by Gino Poulin (University of Manchester, Manchester, UK).

RNAi was performed by feeding and carried out as described previously [52]. Unless stated otherwise, L4 larvae were picked onto plates seeded with bacteria expressing dsRNA corresponding to the gene of interest or containing the control empty vector (EV; containing the L4440 vector with no insert). F1 progeny were picked at the L4 stage and moved to plates seeded with the same bacteria supplemented with 5-fluoro-2′-deoxyuridine (FUDR) (1 mg/ml).

### Tissue-specific RNAi

RNAi deficient *rde-1(ne219)* or *sid-1(pk3321)* mutant strains rescued with tissue-specific *rde-1* or *sid-1* constructs were used for these experiments [30, 31] (see Table S11 for full strain details). RNAi treatment of these strains was performed by feeding and was carried out as previously described [52]. Essentially, five L4-stage hermaphrodites were picked onto plates seeded with bacteria expressing dsRNA corresponding to either control empty vector (EV; L4440) or *utx-1* supplemented with FUDR (1 mg/ml). Each condition was set up such that there were 50–60 worms in total to perform lifespan analyses as described below.

### Lipofuscin-based longevity RNAi screen

RNAi treatment was performed as described above. 30 F1 progeny were picked at the L4 stage and assessed for lipofuscin accumulation at day 3 and day 6 of adulthood (15 worms/time point). Each batch of analysis (around 10 separate RNAi treatments) included the EV control to show changes in the levels of lipofuscin in control animals at the same ages. The gene targets tested in this screen were performed blind to avoid bias. Accumulation of lipofuscin was assessed by eye using a Zeiss AxioSKOP2 microscope, using a 5-point grading system (where − 2 is a substantial decrease compared to the age-matched control, − 1 a small decrease, 0 no change, + 1 a small increase and + 2 a substantial increase). Worms were anaesthetised using 2 μl of 10 μM muscimol (Sigma-Aldrich) and then mounted on 2% agarose pads (as previously described in [49]) for scoring. A Zeiss AxioCamMR digital camera and Axiovision software (release 4.5) were used for digital photography.

### Lifespan and healthspan analysis

Analysis of the lifespan of *C. elegans* strains was performed at 20 °C using the N2 wild-type strain as a control. All strains were fed with OP50 (unless stated otherwise in RNAi experiments). Where appropriate, the OP50 bacterial culture was supplemented with FUDR (1 mg/ml) prior to seeding the plates. Five hermaphrodites were picked onto each plate at the L4 stage (day 0 of the analysis), and they were assessed every other day and scored as dead when unresponsive to three light taps with a platinum wire. In the case of the *utx-1(tm3118)* heterozygous mutants, the strain was not balanced and individual worms were genotyped after death.

Each experiment was set up using at least 60 worms per strain and condition, which usually allowed for the final number of worms scored to be around 50 (after discounting worms that crawled off the plate; see individual figures for n numbers for each experiment). Lifespan analysis was performed on at least two independent biological replicates (repeat data is included in the supplementary tables where appropriate), and experiments were routinely performed blind (where possible) to reduce the chance of operator bias. Unless stated otherwise, all of the lifespan analysis was done in the presence of FUDR.

To analyse age-related motility deficit as a readout of healthspan, worms were picked into ~ 20 μl M9 buffer (42 mM Na_2_HPO_4_, 22 mM KH_2_PO_4_, 86 mM NaCl, 1 mM MgSO_4_) on an unseeded NGM plate. Worms were allowed to acclimatise in the liquid for 30 s. The number of sigmoidal thrashes made by the worm in three sequential 30 s periods was recorded. Ten hermaphrodites per strain per age were analysed, and an average of 30 measurements was used for data comparison.

### Stress survival assays

#### Oxidative stress

L4 worms were plated onto NGM plates (seeded with OP50 or RNAi feeding clones) containing 10 mM Paraquat Dichloride to generate reactive oxygen species (ROS). All plates were maintained at 20 °C. Survival was first assessed at the 22-h time point and every 4 h thereafter (except for an 8 h gap in one of the overnight time points). Worms that were unresponsive to gentle prodding with a platinum wire were scored as dead, and worms that escaped the plates were censored.

#### Heat stress

Day-5 post-L4 worms were placed onto seeded plates before incubating at 35 °C for 6 h. After heating, worms were transferred to freshly seeded plates and maintained at 20 °C. Survival was assessed daily where worms that were unresponsive to gentle prodding with a platinum wire were scored as dead and worms that escaped the plates were censored.

#### UV stress

Day-3 post-L4 worms were transferred onto unseeded plates and allowed to crawl for 10 min to remove excess OP50. The plates were then individually irradiated with 1000 J/m^2^ UVC using a Stratalinker® UV crosslinker. Survival was assessed at least every other day where worms that were unresponsive to gentle prodding with a platinum wire were scored as dead and worms that escaped the plates were censored.

### Generation of *utx-1* and *jmjd-3.2* overexpression constructs

Tissue-specific expression plasmids were constructed using the MultiSite Gateway Three-Fragment Vector Construction Kit (Life Technologies), as previously described (Mariani et al., 2016). *utx-1* cDNA was amplified from cDNA generated from wild-type embryos with the following primers: utx_1fw ATGGACGAATCAGAACCTCT, utx_1rev GGCAGTGAAACTCATCTTATT and cloned into pCR8. Tissue-specific promoters (*dpy-7p and vha-6p*) were cloned into the pDONR P4-P1R vector, whereas *rab-3p and myo-3p* in the pDONR P4-P1R vector, as well as the pDONR P2-RP3 vector containing the *GFP* sequence followed by *unc*-*54* 3′UTR, were a generous gift from Erik Jorgensen. pDEST R4-R3 was used as the final destination vector. Tissue-specific *utx-1* expression constructs were assembled using the Gateway system according to the manufacturer’s instructions to give the following expression constructs: pLSX12(*dpy-7p::utx-1::GFP*), pLSX13(*vha-6p::utx-1::GFP*), pLSX14(*rab-3p::utx-1::GFP*) and pLSX15(*myo-3p::utx-1::GFP*). The generation of the endogenous promoter-driven *utx-1* constructs, *utx-1p::utx-1::GFP* and *utx-1p::utx-1DD::GFP* constructs has been previously described [19].

A similar approach was used to obtain the *jmjd-3.2p::jmjd-3.2::GFP* construct. The *jmjd-3.2* promoter was cloned into the pDONR P4-P1R vector using the following primers: f23_fw GGGGACAACTTTGTATAGAAAAGTTGataaggtcaaatgttaagcctcag, f23_rev GGGGACTGCTTTTTTGTACAAACTTGcttttgaaaaaatcctgaaaaaattatt. The *jmjd-3.2* coding sequence was amplified from cDNA generated from wild-type embryos using the following primers: f23_fw ATGGATAGCGGTGGCCAAGG, f23_rev GGACACCCATTTAAACTCGTCAA and cloned in pCR8. Again, three-factor Gateway construction (adding in the GFP plus *unc-54* 3′UTR sequence detailed above) yielded pLSX10(*jmjd-3.2p::jmjd-3.2::GFP*).

For the *jmjd-3.2p::jmjd-3.2DD::GFP* construct, the *jmjd-3.2 in pCR8* construct was mutated using the QuikChange Site-Directed Mutagenesis Kit (Stratagene). Specifically, the DNA sequence was mutated so that the histidine at position 713 (H713) and the glutamic acid at position 715 (E715) were changed to alanine. The primers: f23_DD_fw ACCACAGCTGCCCTGGCCAACCAAGCTCTCGGGTCC and f23_DD_rv AGCTTGGTTGGCCAGGGCAGCTGTGGTTCTTGCTCC were used for the site-directed mutagenesis to generate the *JMJD-3.2* catalytically dead mutant construct, pLSX03(*jmjd-3.2DD*), which was subsequently used for a three-factor Gateway construction as described above to yield pLSX11(*jmjd-3.2p::jmjd-3.2DD::GFP*).

The DNA sequences of all constructs were verified by sequencing.

### Microinjection and production of transgenic lines

Transgenic *C. elegans* lines were generated using the microinjection method previously described [53, 54]. Prepared plasmid DNA at 10–50 ng/μl was co-injected into wild-type worms with *myo-2p::mCherry* and/or *rol-6*^*+*^ (0.5–5 ng/μl) as injection markers. Several transgenic lines were isolated and analysed for each construct. Transgenic lines in *jmjd-3.2(tm3121)* were generated by crossing. Some transgenic lines for *utx-1* (ZR254-ZR257 and ZR802 and ZR856) are described in [19].

### Real-time quantitative PCR (qRT-PCR)

RNA was isolated from a whole plate of cultured worms, or a minimum of 100 synchronised worms using TRIzol (Invitrogen) and Phase Lock Gel Heavy tubes (5 Prime), followed by chloroform and ethanol RNA extraction.

The reverse transcriptase step was performed using the SuperScript VILO kit (Invitrogen) following the manufacturer’s instructions and using 1 μg of RNA from the RNA extraction protocol. qPCR was performed using SYBR green and Applied Biosystems kits, using the StepOne equipment. Primers used for each gene are available upon request.

Relative quantification of the transcript abundance was calculated using the ΔΔCt method [55]. Every experiment was performed in three technical replicates as well as in three independent biological replicates.

### Microscopy

Worm microscopy was carried out using a Zeiss AxioSKOP2 microscope fitted with differential interference contrast (DIC) and fluorescence optics. Worms were anaesthetised using 2 μL of 10 μM muscimol (Sigma-Aldrich) and then mounted on 2% agarose pads (as previously described in Sulston and Hodgkin, 1988). A Zeiss AxioCamMR digital camera and Axiovision software (release 4.5) were used for digital photography, using a Zeiss × 63 oil immersion objective for tissue analysis, and a Zeiss × 20 objective for whole worm microscopy.

### Statistical methods

The Prism 6 software was used to analyse lifespan data and to perform statistical analysis. Log rank test *p* values were used to assess the statistical significance of the lifespan change (****p < 0.0001, ***p < 0.001, **p < 0.01, *p < 0.05). *p* values and other relevant comparisons (e.g. *n* values, mean, median, maximum values and % change) are presented in figure legends and in Additional files as appropriate. Prism 6 was also used for statistical analysis of brood size and thrashing data using 2-way ANOVA and Dunnett’s multiple comparisons test.

## Supplementary Information

**Additional file 1: Figure S1.** Examples of changes in lipofuscin accumulation after exposure to RNAi feeding clones. 30 F1 progeny were picked at the L4 stage and assessed for lipofuscin accumulation at day 3 and day 6 of adulthood (15 worms/time point). Each set of worms analysed included the EV control to show the changes in lipofuscin levels at comparable ages. Accumulation of lipofuscin was assessed by eye in anaesthetised worms using a Zeiss AxioSKOP2 microscope, using the severity grading system detailed in the methods section.

**Additional file 2: Table S1.** Lifespan analysis of worms subjected to RNAi by feeding clones identified in and inspired by the primary lipofuscin screen. Lifespan was monitored following RNAi of the indicated gene. Average lifespan (in days) is shown, together with relevant *p* values compared with the control. In the case of *cbp-1*, where RNAi resulted in larval lethality of F1s, first generation P0 L4 worms were picked onto RNAi plates containing FUDR, and these were scored for lifespan extension. In the case of *mes-3* and *mes-6* RNAi, 2nd generation F2 worms were scored for lifespan extension. ****p<0.0001, ***p<0.001,**p<0.01,*p<0.05, ns=not significant. EV= Empty Vector control (i.e. worms fed HT115 bacteria transformed with L4440 RNAi vector lacking a genomic insert). At least 60 animals were analysed for each condition.

**Additional file 3: Table S2.** Statistical analysis of lifespan data relating to Fig. 1. Full statistical analysis of lifespan data from Fig. 1 (****p<0.0001, ***p<0.001,**p<0.01,*p<0.05, ns=not significant). Rep = repeat.

**Additional file 4: Figure S2.** Lifespan analysis of *jmjd-3* mutants. The lifespan of *jmjd-3.2* animals was comparable to that of the *jmjd-3.1; jmjd-3.2; jmjd-3.3* triple mutants (*p*=0.19). *jmjd-3.2* animals are longer-lived compared to WT animals (*p*=0.01 (*)). See Additional file 5: Table S3 for the full statistical analysis of the lifespan data, including repeats.

**Additional file 5: Table S3.** Statistical analysis of lifespan data relating to Figure S2. Full statistical analysis of lifespan data from Fig. S2 (****p<0.0001,***p<0.001,**p<0.01,*p<0.05, ns=not significant). EV = empty vector control. Rep = repeat.

**Additional file 6: Figure S3.** Developmental rates of *mes-2*, *jmjd-3.2* and *utx-1* mutant alleles. Gravid adults of each genotype were allowed to lay eggs for 1 hour before being removed from NGM plates seeded with OP50. The resulting progeny and their corresponding developmental stage were recorded after 72 hours at 20oC. *n* >60 animals for each strain.

**Additional file 7: Table S4.** Statistical analysis of lifespan data relating to Fig. 2. Full statistical analysis of lifespan data from Fig. 2 (****p<0.0001,***p<0.001,**p<0.01,*p<0.05, ns=not significant). #consistent with data reported in [14, 15]. EV = empty vector control. Rep = repeat.

**Additional file 8: Figure S4.** DAF-16::GFP translocation following *mes-2*, *jmjd-3.2* and *utx-1* RNAi. Nuclear translocation of *daf-16* using a translational GFP reporter (strain TJ356) was visualised in worms subjected to *mes-2*, *jmjd-3.2*, *utx-1* and *daf-2* RNAi. Worms were placed on RNAi plates at the L4 stage at 20oC and their progeny imaged using fluorescence microscopy when they had reached the third day of adulthood (n=at least 160 animals for each condition). Worms were divided into three categories, depending on the relative degree of DAF-16::GFP nuclear translocation: “mostly cytoplasmic” (where DAF-16::GFP is primarily localised in the cytoplasm), “partly nuclear” (where DAF-16::GFP is localised in both cytoplasm and nuclei) and “mostly nuclear” (where DAF-16::GFP is primarily localised in nuclei). A representative image for each class is shown in the left-hand panel, and the localisation data is shown in the right-hand panel. We used *daf-2* RNAi as a positive control, as this has previously been shown to cause extensive translocation of DAF-16 to the nucleus [14]. Scale Bar = 100μM.

**Additional file 9: Table S5.** Statistical analysis of lifespan data relating to Fig. 3. Full statistical analysis of lifespan data from Fig. 3 (****p<0.0001,***p<0.001,**p<0.01,*p<0.05, ns=not significant). Rep = repeat.

**Additional file 10: Table S6.** Statistical analysis of lifespan data relating to Fig. 4. Full statistical analysis of survival data from Fig. 4 (****p<0.0001,***p<0.001,**p<0.01,*p<0.05, ns=not significant). EV = empty vector control. Rep = repeat.

**Additional file 11: Table S7.** Statistical analysis of lifespan data relating to Fig. 5. Full statistical analysis of lifespan data from Fig. 5 (****p<0.0001,***p<0.001,**p<0.01,*p<0.05, ns=not significant). #consistent with data reported in [14]. EV = empty vector control. Rep = repeat.

**Additional file 12: Figure S5.** Spatial expression of *jmjd-3.2* and *utx-1.* A-B: Animals overexpressing a full length *jmjd-3.2*::GFP construct in wildtype (A) and *jmjd-3.2(tm3121)* mutants (B) were visualised by epifluorescence microscopy and showed spatial expression in a specific subset of neurons (white arrows). C-D: Animals overexpressing full length *utx-1::GFP* in wildtype (C) and *utx-1(tm3118)* mutant background (D) both showed ubiquitous expression (white arrows). E-H: The expression of demethylase dead *jmjd-3.2* (E-F) and *utx-1* (G-H) constructs showed a very similar pattern to the non-mutated constructs. I-J: Whole worm images of animals overexpressing *utx-1::GFP* (I) and *utx-1DD::GFP* (J) show ubiquitous expression of *utx-1*. Scale Bar (A-H) = 10μM; Scale Bar (I-J) = 20μM.

**Additional file 13: Table S8.** Statistical analysis of lifespan data relating to Fig. 6. Full statistical analysis of lifespan data from Fig. 6 (****p<0.0001,***p<0.001,**p<0.01,*p<0.05, ns=not significant). EV = empty vector control. Rep = repeat. Mus = muscle, epi = epidermal, neu = neuronal, int = intestinal.

**Additional file 14: Figure S6.** *daf-16* dependence of lifespan extension due to tissue-specific knockdown or overexpression of *utx-1.* A-B: Lifespan assays were performed on animals tissue-specifically overexpressing *utx-1* in the intestine (A) or neurons (B) subjected to *daf-16* RNAi. Overexpression of *utx-1* gave a moderate lifespan extension in both of these *utx-1* overexpressing strains subjected to *daf-16* RNAi (*p*=0.0001 (***) intestinal; *p*=0.0002 (***) neuronal), suggesting partial *daf-16* independence in each case. N2 and *daf-16* (RNAi) controls are shared between experiments A and B (as the experiments were performed as a large set) although the graphs are separated for clarity. C-F: Lifespan assays were performed on wild type and *daf-16(mu86)* animals subjected to global (C) as well as epidermis (D), neuron (E) and intestine-specific (F) knockdown of *utx-1.* Knockdown of *utx-1* in the wild type background in all cases caused lifespan extension, whereas this was largely abrogated in *daf-16(mu86)* mutants. EV= Empty Vector control (i.e. worms fed HT115 bacteria transformed with L4440 RNAi vector lacking a genomic insert). See Additional file 15: Table S9 for full statistical analysis of lifespan data, including repeats.

**Additional file 15: Table S9.** Statistical analysis of lifespan data relating to Figure S6. Full statistical analysis of lifespan data from Fig. S6 (****p<0.0001,***p<0.001,**p<0.01,*p<0.05, ns=not significant). EV = empty vector control. Rep = repeat. Note some of the data corresponds to data in Fig. 6 (Table S8) as these values were part of the same experiment, split off for clarity.

**Additional file 16: Figure S7.** Knockdown of *daf-2* enhances longevity in a tissue-specific manner. A: Global depletion of *daf-2* was achieved through RNAi and promoted lifespan extension in wild-type animals compared to EV controls (p<0.0001 (****)). B-E: Tissue-specific depletion of *daf-2* enhanced worm longevity when knocked down in the epidermis (B), intestine (C) and neurons (D) (p<0.0001 (****) in all cases), but not in the muscle (E) (*p*=0.82 (ns)), the same tissues implicated in improved longevity mediated by *utx-1* knockdown. EV= Empty Vector control (i.e. worms fed HT115 bacteria transformed with L4440 RNAi vector lacking a genomic insert). See Additional file 17: Table S10 for full statistical analysis of lifespan data.

**Additional file 17: Table S10.** Statistical analysis of lifespan data relating to Figure S7. Full statistical analysis of lifespan data from Fig. S7 (****p<0.0001,***p<0.001,**p<0.01,*p<0.05, ns=not significant). EV = empty vector control. Epi = epidermal, int = intestinal neu = neuronal, mus = muscle.

**Additional file 18: Table S11.** List of *C. elegans* strains used in this study. Strain names are indicated along with genotypes, plasmids and short names used in this report.

## Data Availability

All the data generated or analysed during this study are included in this published article and its supplementary information files.
