# Supplementary material for: H3K27 modifiers regulate lifespan in C. elegans in a context-dependent manner
Source: BMC Biol. 2021 Mar 25;19:59. doi: 10.1186/s12915-021-00984-8 (PMC7995591; doi:10.1186/s12915-021-00984-8)
Supplement: Supplementary file 1 — Additional file 1: Figure S1. Examples of changes in lipofuscin accumulation after exposure to RNAi feeding clones. 30 F1 progeny were picked at the L4 stage and assessed for lipofuscin accumulation at day 3 and day 6 of adulthood (15 worms/time point). Each set of worms analysed included the EV control to show the changes in lipofuscin levels at comparable ages. Accumulation of lipofuscin was assessed by eye in anaesthetised worms using a Zeiss AxioSKOP2 microscope, using the severity grading system detailed in the methods section. [file 12915_2021_984_MOESM1_ESM.pdf]

Fig. S1

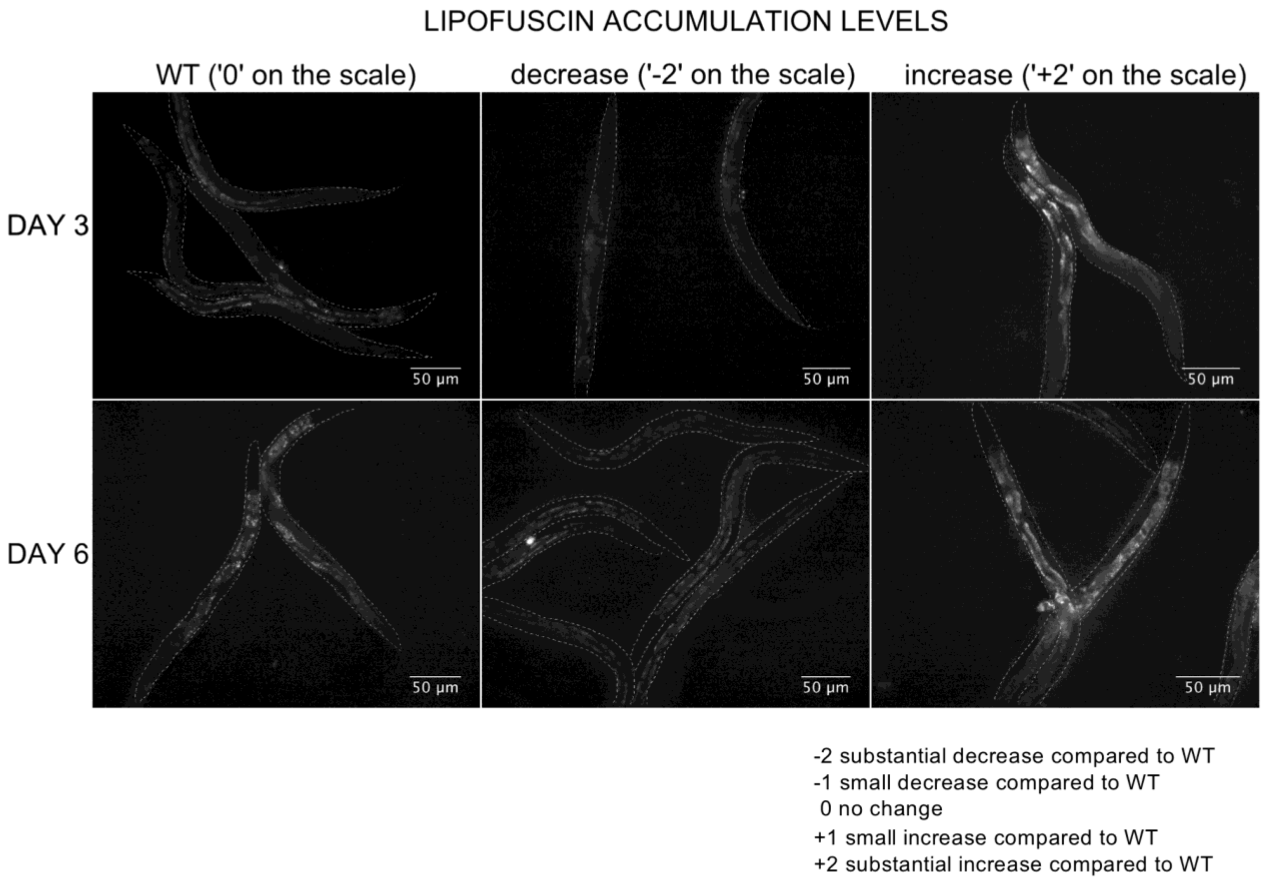

Figure S1. Examples of changes in lipofuscin accumulation after exposure to RNAi feeding clones

30 F1 progeny were picked at the L4 stage and assessed for lipofuscin accumulation at day 3 and day 6 of adulthood (15 worms/time point). Each set of worms analysed included the EV control to show the changes in lipofuscin levels at comparable ages. Accumulation of lipofuscin was assessed by eye in anaesthetised worms using a Zeiss AxioSKOP2 microscope, using the severity grading system detailed in the methods section.
