# Supplementary material for: H3K27 modifiers regulate lifespan in C. elegans in a context-dependent manner
Source: BMC Biol. 2021 Mar 25;19:59. doi: 10.1186/s12915-021-00984-8 (PMC7995591; doi:10.1186/s12915-021-00984-8)
Supplement: Supplementary file 2 — Additional file 2: Table S1. Lifespan analysis of worms subjected to RNAi by feeding clones identified in and inspired by the primary lipofuscin screen. Lifespan was monitored following RNAi of the indicated gene. Average lifespan (in days) is shown, together with relevant p values compared with the control. In the case of cbp-1, where RNAi resulted in larval lethality of F1s, first generation P0 L4 worms were picked onto RNAi plates containing FUDR, and these were scored for lifespan extension. In the case of mes-3 and mes-6 RNAi, 2nd generation F2 worms were scored for lifespan extension. ****p<0.0001, ***p<0.001,**p<0.01,*p<0.05, ns=not significant. EV= Empty Vector control (i.e. worms fed HT115 bacteria transformed with L4440 RNAi vector lacking a genomic insert). At least 60 animals were analysed for each condition. [file 12915_2021_984_MOESM2_ESM.pdf]

Table S1

RNAi clone	median survival	maximum lifespan	Log Rank Test *p* value relative to EV control
**EV**	15	28	
mes-2	17	33	0.03 (*)
jmjd-3.2	18	33	0.007 (**)
cbp-1 (post larval)	18	27	0.008 (**)
isw-1	18	34	0.003 (**)
**EV**	15	25	
mes-3	20	35	<0.0001 (****)
mes-6	20	31	<0.0001 (****)
**EV**	18	28	
utx-1	20	32	0.003 (**)
EV	13	25	
jmjd-3.1	13	21	0.43 (ns)
jmjd-3.3	11	25	0.28 (ns)

Table S1. Lifespan analysis of worms subjected to RNAi by feeding clones identified in and inspired by the primary lipofuscin screen

Lifespan was monitored following RNAi of the indicated gene. Average lifespan (in days) is shown, together with relevant *p* values compared with the control. In the case of *cbp-1*, where RNAi resulted in larval lethality of F1s, first generation P0 L4 worms were picked onto RNAi plates containing FUDR, and these were scored for lifespan extension. In the case of *mes-3* and *mes-6* RNAi, 2nd generation F2 worms were scored for lifespan extension. ****p<0.0001, ***p<0.001,**p<0.01,*p<0.05, ns=not significant. EV= Empty Vector control (*i.e.* worms fed HT115 bacteria transformed with L4440 RNAi vector lacking a genomic insert). At least 60 animals were analysed for each condition.
