# Supplementary material for: H3K27 modifiers regulate lifespan in C. elegans in a context-dependent manner
Source: BMC Biol. 2021 Mar 25;19:59. doi: 10.1186/s12915-021-00984-8 (PMC7995591; doi:10.1186/s12915-021-00984-8)
Supplement: Supplementary file 3 — Additional file 3: Table S2. Statistical analysis of lifespan data relating to Fig. 1. Full statistical analysis of lifespan data from Fig. 1 (****p<0.0001, ***p<0.001,**p<0.01,*p<0.05, ns=not significant). Rep = repeat. [file 12915_2021_984_MOESM3_ESM.pdf]

Table S2

Fig ref	Strain / condition	no. of animals	mean lifespan	% lifespan change (vs control)	median lifespan	maximum lifespan	Log Rank Test *p* value relative to control
**1B**	N2 control	46	17.6		18	26	
	mes-2(tm5007)	47	21.2	20% increase	22	30	0.0003 (***)
	mes-2(ok2480)	47	28.4	61% increase	30	42	<0.0001 (****)
**1B rep**	N2 control	95	20.5		20	27	
	mes-2(tm5007)	95	22.5	10% increase	22	33	<0.0001 (****)
	N2 control	51	15.6		16	23	
	mes-2(ok2480)	50	21.7	36% increase	21	33	<0.0001 (****)
**1C**	N2 control	55	18.3		18	30	
	SP127 balancer control	54	17.8		18	28	0.4 (ns)
	mes-2(bn11)	46	22.2	21% increase (vs N2)	22	36	<0.0001 (****)
	mes-2(bn11)/+	59	22.6	23% increase (vs N2)	22	38	0.0005 (***) compared with N2
**1C rep**	N2 control	48	15.2		15	27	
	mes-2(bn11)	59	19.6	30% increase (vs N2)	19	35	<0.0001 (****)
	N2 control	95	20.5		20	27	
	mes-2(bn11)/+	79	22.4	9% increase (vs N2)	22	28	<0.0001 (****)
**1E**	N2 control	46	17.6		18	26	
	jmjd-3.2(tm3121)	45	20.5	16% increase	20	32	0.005 (**)
**1E rep**	N2 control	55	18.3		18	30	
	jmjd-3.2(tm3121)	57	20.8	14% increase	20	34	0.012 (*)
**1F**	N2 control	48	14.1		14	22	
	utx-1(tm3118)/+	42	19.4	38% increase	20	34	<0.0001 (****)
**1F rep**	*(see Fig 2C or 3F)*						

Table S2. Statistical analysis of lifespan data relating to Figure 1

Full statistical analysis of lifespan data from Fig. 1 (****p<0.0001,***p<0.001,**p<0.01,*p<0.05, ns=not significant). Rep = repeat.
