# Supplementary material for: H3K27 modifiers regulate lifespan in C. elegans in a context-dependent manner
Source: BMC Biol. 2021 Mar 25;19:59. doi: 10.1186/s12915-021-00984-8 (PMC7995591; doi:10.1186/s12915-021-00984-8)
Supplement: Supplementary file 4 — Additional file 4: Figure S2. Lifespan analysis of jmjd-3 mutants. The lifespan of jmjd-3.2 animals was comparable to that of the jmjd-3.1; jmjd-3.2; jmjd-3.3 triple mutants (p=0.19). jmjd-3.2 animals are longer-lived compared to WT animals (p=0.01 (*)). See Additional file 5: Table S3 for the full statistical analysis of the lifespan data, including repeats. [file 12915_2021_984_MOESM4_ESM.pdf]

Fig. S2

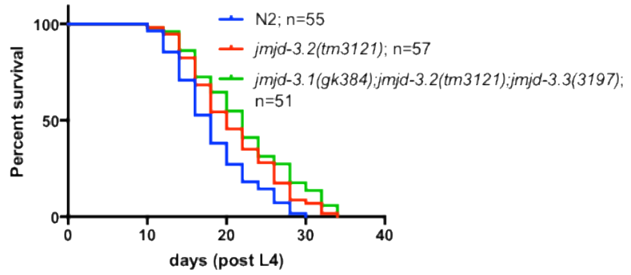

Figure S2. Lifespan analysis of *jmjd-3* mutants

The lifespan of *jmjd-3.2* animals was comparable to that of the *jmjd-3.1; jmjd-3.2; jmjd-3.3* triple mutants (p=0.19). *jmjd-3.2* animals are longer-lived compared to WT animals (p=0.01 (*)). See Additional file 5:Table S3 for the full statistical analysis of the lifespan data, including repeats.
