# Supplementary material for: H3K27 modifiers regulate lifespan in C. elegans in a context-dependent manner
Source: BMC Biol. 2021 Mar 25;19:59. doi: 10.1186/s12915-021-00984-8 (PMC7995591; doi:10.1186/s12915-021-00984-8)
Supplement: Supplementary file 5 — Additional file 5: Table S3. Statistical analysis of lifespan data relating to Figure S2. Full statistical analysis of lifespan data from Fig. S2 (****p<0.0001,***p<0.001,**p<0.01,*p<0.05, ns=not significant). EV = empty vector control. Rep = repeat. [file 12915_2021_984_MOESM5_ESM.pdf]

Table S3

Fig ref	Strain / condition	no. of animals	mean lifespan	% lifespan change (vs control)	median lifespan	maximum lifespan	Log Rank Test *p* value relative to control
**S2**	N2	55	18.3		18	30	
	jmjd-3.2(tm3121)	57	20.8	14% increase	20	34	0.0117 (*)
	jmjd-3.1(gk384); jmjd-3.2(tm3121); jmjd-3.3(tm3197)	51	22.2		22	34	0.19 (ns) compared with *jmjd-3.2(tm3121)*
**S2 rep**	N2	79	14.7		15	21	
	jmjd-3.2(tm3121)	76	16.1	10% increase	16	25	0.003 (**)
	jmjd-3.1(gk384); jmjd-3.2(tm3121); jmjd-3.3(tm3197)	75	15.5		15	27	0.54 (ns) compared with *jmjd-3.2(tm3121)*

Table S3. Statistical analysis of lifespan data relating to Figure S2

Full statistical analysis of lifespan data from Fig. S2 (****p<0.0001,***p<0.001,**p<0.01,*p<0.05, ns=not significant). EV = empty vector control. Rep = repeat.
