# Supplementary material for: H3K27 modifiers regulate lifespan in C. elegans in a context-dependent manner
Source: BMC Biol. 2021 Mar 25;19:59. doi: 10.1186/s12915-021-00984-8 (PMC7995591; doi:10.1186/s12915-021-00984-8)
Supplement: Supplementary file 6 — Additional file 6: Figure S3. Developmental rates of mes-2, jmjd-3.2 and utx-1 mutant alleles. Gravid adults of each genotype were allowed to lay eggs for 1 hour before being removed from NGM plates seeded with OP50. The resulting progeny and their corresponding developmental stage were recorded after 72 hours at 20oC. n >60 animals for each strain. [file 12915_2021_984_MOESM6_ESM.pdf]

Fig. S3

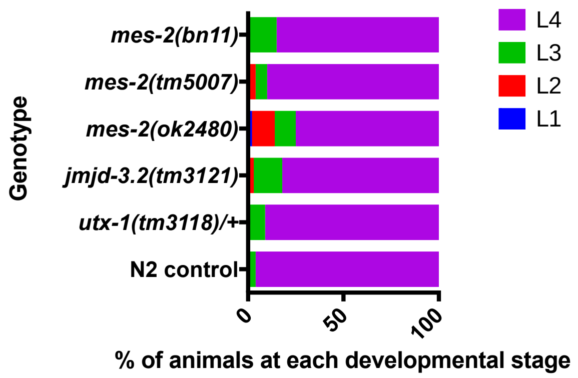

Figure S3. Developmental rates of *mes-2*, *jmjd-3.2* and *utx-1* mutant alleles

Gravid adults of each genotype were allowed to lay eggs for 1 hour before being removed from NGM plates seeded with OP50. The resulting progeny and their corresponding developmental stage were recorded after 72 hours at 20°C. n >60 animals for each strain.
