# Supplementary material for: H3K27 modifiers regulate lifespan in C. elegans in a context-dependent manner
Source: BMC Biol. 2021 Mar 25;19:59. doi: 10.1186/s12915-021-00984-8 (PMC7995591; doi:10.1186/s12915-021-00984-8)
Supplement: Supplementary file 7 — Additional file 7: Table S4. Statistical analysis of lifespan data relating to Fig. 2. Full statistical analysis of lifespan data from Fig. 2 (****p<0.0001,***p<0.001,**p<0.01,*p<0.05, ns=not significant). #consistent with data reported in [14, 15]. EV = empty vector control. Rep = repeat. [file 12915_2021_984_MOESM7_ESM.pdf]

Table S4

Fig ref	Strain / condition	no. of animals	mean lifespan	% lifespan change (vs control)	median lifespan	maximum lifespan	Log Rank Test *p* value relative to control
**2A**	N2 EV control	50	17.2		16	26	
	daf-16 RNAi	56	13.6	21% decrease	14	22	<0.0001 (****)
	mes-2(tm5007) + EV control	51	19.0	11% increase	18	30	0.02 (*)
	mes-2(tm5007) + *daf-16* RNAi	49	14.0	26% decrease (vs mes-2 EV)	14	18	0.94 (ns) compared with *daf-16*<br><0.0001 (****) compared with *mes-2*
**2A rep**	N2 EV control	57	14.3		13	23	
	daf-16 RNAi	56	12.5	13% decrease	13	17	0.0008 (***)
	mes-2(tm5007) + EV control	56	16.1	13% increase	15	30	0.02 (*)
	mes-2(tm5007) + *daf-16* RNAi	57	11.8	27% decrease (vs mes-2 EV)	13	15	0.08 (ns) compared with *daf-16*<br><0.0001 (****) compared with *mes-2*
**2B**	N2 EV control	50	17.2		16	26	
	daf-16 RNAi	57	13.8	20% decrease	14	22	<0.0001 (****)
	jmjd-3.2(tm3121) + EV control	59	18.6	8% increase	18	30	0.04 (*)
	jmjd-3.2(tm3121) + *daf-16* RNAi	54	14.3	23% decrease (vs jmjd-3.2 in EV)	14	22	0.36 (ns) compared with *daf-16*<br><0.0001 (****) compared with *jmjd-3.2*
**2B rep**	N2 EV control	79	14.7		15	21	
	daf-16 RNAi	79	13	12% decrease	13	19	<0.0001 (****)
	jmjd-3.2(tm3121) + EV control	76	16.1	10% increase	16	25	0.003 (**)
	jmjd-3.2(tm3121) + *daf-16* RNAi	79	13.2	18% decrease (vs jmjd-3.2 in EV)	13	20	0.68 (ns) compared with *daf-16*<br><0.0001 (****) compared with *jmjd-3.2*
**2C**[#]	N2 EV control	50	17.1		16	26	
	daf-16 RNAi	57	13.8	19% decrease	14	22	<0.0001 (****)
	utx-1(tm3118)/+ + EV control	52	21.3	25% increase	20	34	<0.0001 (****)
	utx-1(tm3118)/+ + *daf-16* RNAi	46	13.6	36% decrease (vs tm3118 in EV)	14	20	0.58 (ns) compared with *daf-16*

							<0.0001 (****) compared with *utx-1*
**2D**	N2 EV control	49	17.8		18	28	
	N2 + *utx-1* RNAi	55	20.8	17% increase	20	32	0.003 (**)
	jmjd-3.2(tm3121)+ EV	53	19.7	11% increase	20	32	0.03 (*)
	jmjd-3.2(tm3121)+ utx-1 RNAi	56	22.5	14% increase (vs jmjd-3.2 in EV)	22	36	0.007 (**) compared with *jmjd-3.2 EV*
**2D rep**	N2 EV control	79	14.7		15	21	
	N2 + *utx-1* RNAi	79	20.8	41% increase	19	34	<0.0001 (****)
	jmjd-3.2(tm3121)+ EV	76	16.1	10% increase	16	25	0.003 (**)
	jmjd-3.2(tm3121)+ utx-1 RNAi	74	20.3	26% increase (vs jmjd-3.2 in EV)	20	37	<0.0001 (****) compared with *jmjd-3.2 EV*

Table S4. Statistical analysis of lifespan data relating to Fig 2

Full statistical analysis of lifespan data from Fig. 2 (****p<0.0001,***p<0.001,**p<0.01,*p<0.05, ns=not significant). #consistent with data reported in [14,15]. EV = empty vector control. Rep = repeat.
