# Supplementary material for: H3K27 modifiers regulate lifespan in C. elegans in a context-dependent manner
Source: BMC Biol. 2021 Mar 25;19:59. doi: 10.1186/s12915-021-00984-8 (PMC7995591; doi:10.1186/s12915-021-00984-8)
Supplement: Supplementary file 8 — Additional file 8: Figure S4. DAF-16::GFP translocation following mes-2, jmjd-3.2 and utx-1 RNAi. Nuclear translocation of daf-16 using a translational GFP reporter (strain TJ356) was visualised in worms subjected to mes-2, jmjd-3.2, utx-1 and daf-2 RNAi. Worms were placed on RNAi plates at the L4 stage at 20oC and their progeny imaged using fluorescence microscopy when they had reached the third day of adulthood (n=at least 160 animals for each condition). Worms were divided into three categories, depending on the relative degree of DAF-16::GFP nuclear translocation: “mostly cytoplasmic” (where DAF-16::GFP is primarily localised in the cytoplasm), “partly nuclear” (where DAF-16::GFP is localised in both cytoplasm and nuclei) and “mostly nuclear” (where DAF-16::GFP is primarily localised in nuclei). A representative image for each class is shown in the left-hand panel, and the localisation data is shown in the right-hand panel. We used daf-2 RNAi as a positive control, as this has previously been shown to cause extensive translocation of DAF-16 to the nucleus [14]. Scale Bar = 100μM. [file 12915_2021_984_MOESM8_ESM.pdf]

Fig. S4

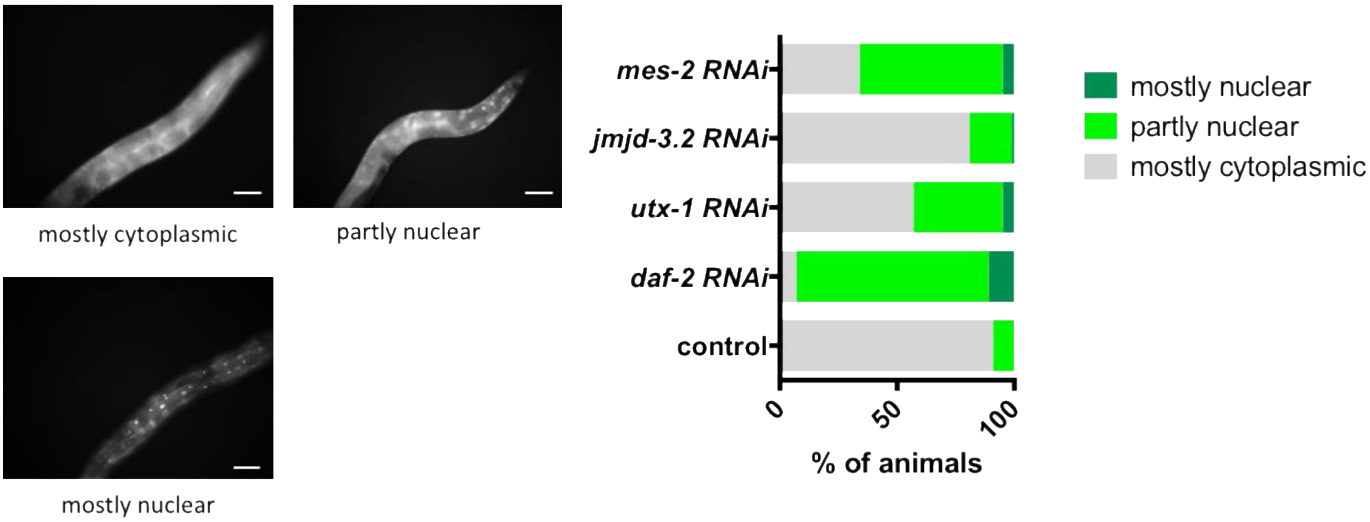

Figure S4. DAF-16::GFP translocation following *mes-2*, *jmjd-3.2* and *utx-1* RNAi

Nuclear translocation of *daf-16* using a translational GFP reporter (strain TJ356) was visualised in worms subjected to *mes-2*, *jmjd-3.2*, *utx-1* and *daf-2* RNAi. Worms were placed on RNAi plates at the L4 stage at 20°C and their progeny imaged using fluorescence microscopy when they had reached the third day of adulthood (n=at least 160 animals for each condition). Worms were divided into three categories, depending on the relative degree of DAF-16::GFP nuclear translocation: “mostly cytoplasmic” (where DAF-16::GFP is primarily localised in the cytoplasm), “partly nuclear” (where DAF-16::GFP is localised in both cytoplasm and nuclei) and “mostly nuclear” (where DAF-16::GFP is primarily localised in nuclei). A representative image for each class is shown in the left-hand panel, and the localisation data is shown in the right-hand panel. We used *daf-2* RNAi as a positive control, as this has previously been shown to cause extensive translocation of DAF-16 to the nucleus [14]. Scale Bar = 100μM.
