# Supplementary material for: H3K27 modifiers regulate lifespan in C. elegans in a context-dependent manner
Source: BMC Biol. 2021 Mar 25;19:59. doi: 10.1186/s12915-021-00984-8 (PMC7995591; doi:10.1186/s12915-021-00984-8)
Supplement: Supplementary file 9 — Additional file 9: Table S5. Statistical analysis of lifespan data relating to Fig. 3. Full statistical analysis of lifespan data from Fig. 3 (****p<0.0001,***p<0.001,**p<0.01,*p<0.05, ns=not significant). Rep = repeat. [file 12915_2021_984_MOESM9_ESM.pdf]

Table S5

Fig ref	Strain / condition	no. of animals	mean lifespan	% lifespan change (vs control)	median lifespan	maximum lifespan	Log Rank Test *p* value relative to control
**3C**	N2 control	59	16.5		16	24	
	N2 + *jmjd-3.2 OE*	55	21.0	27% increase	20	36	<0.0001 (****)
	N2 + *jmjd-3.2DD OE*	59	22.0	33% increase	22	34	<0.0001 (****) compared with *N2 control*<br>0.57 (ns) compared with *jmjd-3.2 OE*
**3C rep**	N2 control	53	13.9		14	22	
	N2 + *jmjd-3.2 OE*	50	20.7	49% increase	20	32	<0.0001 (****)
	N2 + *jmjd-3.2DD OE*	53	19.8	42% increase	20	32	<0.0001 (****) compared with *N2 control*<br>0.61 (ns) compared with *jmjd-3.2 OE*
**3D**	N2 control	55	18.3		18	30	
	N2 + *utx-1 OE*	49	23.1	26% increase	22	38	<0.0001 (****)
	N2 + *utx-1DD OE*	54	17	26% decrease (vs utx-1 OE)	18	28	0.11 (ns) compared with *N2 control*<br><0.0001 (****) compared with *utx-1 OE*
**3D rep**	N2 control	52	17.5		16	28	
	N2 + *utx-1 OE*	58	20.6	18% increase	20	36	0.0013 (**)
	N2 + *utx-1DD OE*	57	17.1	17% decrease (vs utx-1 OE)	16	26	0.58 (ns) compared with *N2 control*<br>0.0002 (***) compared with *utx-1 OE*
**3E**	N2 control	99	15.6		16	26	
	jmjd-3.2(tm3121)	45	20.2	29% increase	20	32	<0.0001 (****)
	jmjd-3.2(tm3121)+ jmjd-3.2 OE	53	18.3	17% increase (vs N2)	18	28	0.0007 (***) compared with *N2 control*<br>0.09 (ns)

							compared with *jmjd-3.2(tm3121)*
	jmjd-3.2(tm3121)+ jmjd-3.2DD OE	58	18.1	16% increase (vs N2)	18	30	0.0008 (***) compared with *N2 control* 0.14 (ns) compared with *jmjd-3.2(tm3121)* 0.81 (ns) compared with *jmjd-3.2(tm3121) + jmjd-3.2 OE*
**3F**	N2 control	48	14.9		14	22	
	utx-1(tm3118)/+	42	21.0	41% increase	20	34	<0.0001 (****)
	utx-1(tm3118) + utx-1 OE	44	29.6	99% increase (vs N2) 41% increase (vs utx-1(tm3118/+))	30	50	<0.0001 (****) compared with *N2 control* <0.0001 (****) compared with *utx-1(tm3118)/+*
	utx-1(tm3118) + utx-1DD OE	44	20.1	35% increase (vs N2) 32% decrease (vs utx-1(tm3118)+utx-1 OE)	19	34	<0.0001 (****) compared with *N2 control* 0.49 (ns) compared with *utx-1(tm3118)/+* <0.0001 (****) compared with *utx-1(tm3118) + utx-1 OE*
**3F rep**	N2 control	56	14.7		14	24	
	utx-1(tm3118)/+	46	16.9	15% increase	16	30	0.0067 (**)
	utx-1(tm3118) + utx-1 OE	46	21.3	45% increase (vs N2) 26% increase (vs utx-1(tm3118)/+)	18	38	<0.0001 (****) compared with *N2 control* <0.0001 (****) compared with *utx-1(tm3118)/+*
	utx-1(tm3118) + utx-1DD OE	54	17.8	21% increase (vs N2) 16% decrease (vs utx-1(tm3118)+utx-1 OE)	18	30	0.0001 (***) compared with *N2 control* 0.47 (ns) compared with *utx-1(tm3118)/+* 0.0041 (**) compared with *utx-1(tm3118) + utx-1 OE*

Table S5. Statistical analysis of lifespan data relating to Figure 3

Full statistical analysis of lifespan data from Fig. 3 (****$p<0.0001$,***$p<0.001$,**$p<0.01$,*$p<0.05$, ns=not significant). Rep = repeat.
