# Supplementary material for: H3K27 modifiers regulate lifespan in C. elegans in a context-dependent manner
Source: BMC Biol. 2021 Mar 25;19:59. doi: 10.1186/s12915-021-00984-8 (PMC7995591; doi:10.1186/s12915-021-00984-8)
Supplement: Supplementary file 10 — Additional file 10: Table S6. Statistical analysis of lifespan data relating to Fig. 4. Full statistical analysis of survival data from Fig. 4 (****p<0.0001,***p<0.001,**p<0.01,*p<0.05, ns=not significant). EV = empty vector control. Rep = repeat. [file 12915_2021_984_MOESM10_ESM.pdf]

**Table S6**

| Fig ref | Strain / condition | no. of animals | mean survival | % survival change (vs control) | median survival | maximum survival | Log Rank Test *p* value relative to control |
|---|---|---|---|---|---|---|---|
| **4A** | N2 control + 10mM paraquat | 47 | 49 hours | | 52 hours | 72 hours | <0.0001 (****) |
| | *utx-1 OE* + 10mM paraquat | 49 | 61 hours | 25% increase | 59 hours | 95 hours | |
| **4A rep** | N2 control + 10mM paraquat | 50 | 49 hours | | 50 hours | 66 hours | 0.0212 (*) |
| | *utx-1 OE* + 10mM paraquat | 49 | 57 hours | 16% increase | 61 hours | 89 hours | |
| **4B** | N2 control + UV (1000Jm$^{-2}$) | 40 | 4.6 | | 5 | 9 | |
| | *utx-1 OE* + UV (1000Jm$^{-2}$) | 45 | 6.5 | 41% increase | 7 | 11 | <0.0001 (****) |
| **4B rep** | N2 control + UV (1000Jm$^{-2}$) | 55 | 3.9 | | 4 | 7 | |
| | *utx-1 OE* + UV (1000Jm$^{-2}$) | 54 | 5.4 | 38% increase | 6 | 10 | <0.0001 (****) |
| **4C** | N2 control + 35$^{o}$C acute heat stress | 40 | 6.9 | | 7.5 | 12 | |
| | *utx-1 OE* + 35$^{o}$C acute heat stress | 45 | 18 | 161% increase | 20 | 30 | <0.0001 (****) |

**Table S6. Statistical analysis of lifespan data relating to Figure 4**

Full statistical analysis of survival data from Fig. 4 (****p<0.0001,***p<0.001,**p<0.01,*p<0.05, ns=not significant). EV = empty vector control. Rep = repeat.
