# Supplementary material for: H3K27 modifiers regulate lifespan in C. elegans in a context-dependent manner
Source: BMC Biol. 2021 Mar 25;19:59. doi: 10.1186/s12915-021-00984-8 (PMC7995591; doi:10.1186/s12915-021-00984-8)
Supplement: Supplementary file 11 — Additional file 11: Table S7. Statistical analysis of lifespan data relating to Fig. 5. Full statistical analysis of lifespan data from Fig. 5 (****p<0.0001,***p<0.001,**p<0.01,*p<0.05, ns=not significant). #consistent with data reported in [14]. EV = empty vector control. Rep = repeat. [file 12915_2021_984_MOESM11_ESM.pdf]

Table S7

Fig ref	Strain / condition	no. of animals	mean lifespan	% lifespan change (vs control)	median lifespan	maximum lifespan	Log Rank Test *p* value relative to control
**5A**	N2 + EV control	52	17.5		16	28	
	N2 + *daf-16* RNAi	57	13.8	21% decrease	14	22	<0.0001 (****) compared with *N2 control*
	utx-1(tm3118) + *utx-1 OE* + EV control	58	31.3	79% increase	30	46	<0.0001 (****) compared with *N2 control*
	utx-1(tm3118) + *utx-1 OE* + *daf-16* RNAi	58	17.0	23% increase (vs N2 in daf-16 RNAi) 46% decrease (vs utx-1 OE in daf-16 RNAi)	16	24	<0.0001 (****) compared with *daf-16 RNAi* <0.0001 (****) compared with *utx-1(tm3118)+utx-1 OE*
**5A rep**	N2 + EV control	54	16.3		16	29	
	N2 + *daf-16* RNAi	56	13.3	18% decrease	14	17	<0.0001 (****) compared with *N2 control*
	utx-1(tm3118) + *utx-1 OE* + EV control	57	30.0	84% increase	31	47	<0.0001 (****) compared with *N2 control*
	utx-1(tm3118) + *utx-1 OE* + *daf-16* RNAi	53	14.2	7% increase (vs N2 in daf-16 RNAi) 53% decrease (vs utx-1 OE in daf-16 RNAi)	14	19	0.0005 (***) compared with *daf-16 RNAi* <0.0001 (****) compared with *utx-1(tm3118)+utx-1 OE*
**5B** #	N2 + EV control	56	14.5		14	27	
	N2 + *utx-1* RNAi	56	19	31% increase	19	28	<0.0001 (****)
	daf-2(e1370) + EV control	55	38.9	168% increase (vs N2)	42	61	<0.0001 (****) compared with N2
	daf-2(e1370) + utx-1 RNAi	58	39.1	170% increase (vs N2)	42	56	<0.0001 (****) compared with N2 0.45 (ns) compared with *daf-2(e1370)*
**5C**	N2	58	17.2		19	28	
	utx-1(tm3118) + utx-1 OE	56	27.9	62% increase	32	42	<0.0001 (****) compared with N2
	daf-2(e1370)	58	36.1	110% increase (vs N2) 29% increase (vs utx-1(tm3118) + utx-1OE)	39	53	<0.0001 (****) compared with N2 <0.0001 (****) compared with utx-1(tm3118) + utx-1OE
	daf-2(e1370); utx-1(tm3118) + utx-1 OE	60	40.7	46% increase (vs utx-1(tm3118) + utx-1OE) 13% increase (vs daf-2(e1370))	47	61	<0.0001 (****) compared with utx-1(tm3118) + utx-1OE 0.0023 (**) compared with daf-2(e1370)
**5C rep**	N2	47	15.7		17	23	
	utx-1(tm3118) + utx-1 OE	46	20.1	28% increase	21	35	<0.0001 (****) compared with N2

daf-2(e1370)	59	33.0	110% increase (vs N2) 64% increase (vs utx-1(tm3118) + utx-1OE)	32	48	<0.0001 (****) compared with N2 <0.0001 (****) compared with utx-1(tm3118) + utx-1OE
daf-2(e1370); utx-1(tm3118) + utx-1 OE	52	42.0	109% increase (vs utx-1(tm3118) + utx-1OE) 27% increase (vs daf-2(e1370))	42	62	<0.0001 (****) compared with utx-1(tm3118) + utx-1OE <0.0001 (****) compared with daf-2(e1370)

Table S7. Statistical analysis of lifespan data relating to Figure 5

Full statistical analysis of lifespan data from Fig. 5 (****p<0.0001,***p<0.001,**p<0.01,*p<0.05, ns=not significant). #consistent with data reported in [14]. EV = empty vector control. Rep = repeat.
