# Supplementary material for: H3K27 modifiers regulate lifespan in C. elegans in a context-dependent manner
Source: BMC Biol. 2021 Mar 25;19:59. doi: 10.1186/s12915-021-00984-8 (PMC7995591; doi:10.1186/s12915-021-00984-8)
Supplement: Supplementary file 12 — Additional file 12: Figure S5. Spatial expression of jmjd-3.2 and utx-1. A-B: Animals overexpressing a full length jmjd-3.2::GFP construct in wildtype (A) and jmjd-3.2(tm3121) mutants (B) were visualised by epifluorescence microscopy and showed spatial expression in a specific subset of neurons (white arrows). C-D: Animals overexpressing full length utx-1::GFP in wildtype (C) and utx-1(tm3118) mutant background (D) both showed ubiquitous expression (white arrows). E-H: The expression of demethylase dead jmjd-3.2 (E-F) and utx-1 (G-H) constructs showed a very similar pattern to the non-mutated constructs. I-J: Whole worm images of animals overexpressing utx-1::GFP (I) and utx-1DD::GFP (J) show ubiquitous expression of utx-1. Scale Bar (A-H) = 10μM; Scale Bar (I-J) = 20μM. [file 12915_2021_984_MOESM12_ESM.pdf]

**Fig. S5**

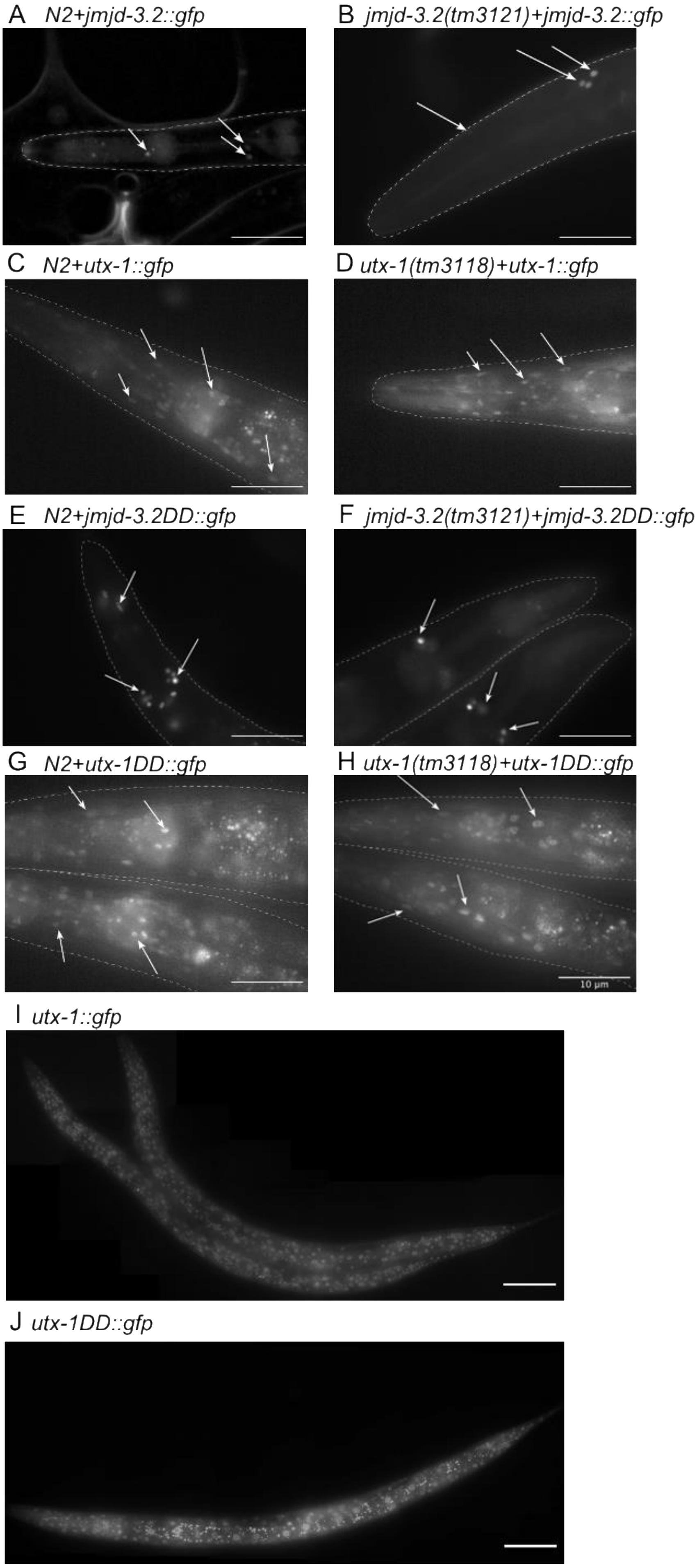
A N2+jmjd-3.2::gfp
B jmjd-3.2(tm3121)+jmjd-3.2::gfp
C N2+utx-1::gfp
D utx-1(tm3118)+utx-1::gfp
E N2+jmjd-3.2DD::gfp
F jmjd-3.2(tm3121)+jmjd-3.2DD::gfp
G N2+utx-1DD::gfp
H utx-1(tm3118)+utx-1DD::gfp
10 μm
I utx-1::gfp
J utx-1DD::gfp

**Figure S5. Spatial expression of *jmjd-3.2* and *utx-1***

A-B: Animals overexpressing a full length *jmjd-3.2*::GFP construct in wildtype (A) and *jmjd-3.2(tm3121)* mutants (B) were visualised by epifluorescence microscopy and showed spatial expression in a specific subset of neurons (white arrows). C-D: Animals overexpressing full length *utx-1::GFP* in wildtype (C) and *utx-1(tm3118)* mutant background (D) both showed ubiquitous expression (white arrows). E-H: The expression of demethylase dead *jmjd-3.2* (E-F) and *utx-1* (G-H) constructs showed a very similar pattern to the non-mutated constructs. I-J: Whole worm images of animals overexpressing *utx-1::GFP* (I) and *utx-1DD::GFP* (J) show ubiquitous expression of *utx-1*. Scale Bar (A-H) = 10µM; Scale Bar (I-J) = 20µM.
