# Supplementary material for: H3K27 modifiers regulate lifespan in C. elegans in a context-dependent manner
Source: BMC Biol. 2021 Mar 25;19:59. doi: 10.1186/s12915-021-00984-8 (PMC7995591; doi:10.1186/s12915-021-00984-8)
Supplement: Supplementary file 13 — Additional file 13: Table S8. Statistical analysis of lifespan data relating to Fig. 6. Full statistical analysis of lifespan data from Fig. 6 (****p<0.0001,***p<0.001,**p<0.01,*p<0.05, ns=not significant). EV = empty vector control. Rep = repeat. Mus = muscle, epi = epidermal, neu = neuronal, int = intestinal. [file 12915_2021_984_MOESM13_ESM.pdf]

Table S8

Fig ref	Strain / condition	no. of animals	mean lifespan	% lifespan change (vs control)	median lifespan	maximum lifespan	Log Rank Test *p* value relative to control
**6B**	N2 control	59	16.5		16	24	
	myo-3p::utx-1::GFP (muscle)	53	15.1		14	30	0.14 (ns)
	dpy-7p::utx-1::GFP (epidermis)	42	17.1		16	26	0.27 (ns)
	rab-3p::utx-1::GFP (neuron)	55	22.8	38% increase	22	34	<0.0001 (****)
	vha-6p::utx-1::GFP (intestine)	58	21.7	32% increase	22	34	<0.0001 (****)
**6B rep**	*see Fig S6A-B*						
**6C (mus)**	NR350 + EV RNAi	37	13.7		14	20	
	NR350 + *utx-1* RNAi	43	13.7		14	18	0.96 (ns)
**6C rep**	NR350 + EV RNAi	47	13.6		14	22	
	NR350 + *utx-1* RNAi	48	13.3		14	20	0.54 (ns)
**6D (epi)**	NR222 + EV RNAi	44	14.4		14	24	
	NR222 + *utx-1* RNAi	43	19.4	35% increase	20	28	<0.0001 (****)
**6D rep**	NR222 + EV RNAi	55	15.8		16	25	
	NR222 + *utx-1* RNAi	59	20.2	28% increase	21	27	<0.0001 (****)
**6E (neu)**	TU3401 + EV RNAi	48	15.1		14	20	
	TU3401 + *utx-1* RNAi	48	21.8	44% increase	22	29	<0.0001 (****)
**6E rep**	TU3401 + EV RNAi	56	16.1		16	21	
	TU3401 + *utx-1* RNAi	58	22.8	42% increase	24	30	<0.0001 (****)
**6F (int)**	VP303 + EV RNAi	51	14.7		14	24	
	VP303 + *utx-1* RNAi	56	16.6	13% increase	16	24	0.009 (**)
**6F rep**	VP303 + EV RNAi	42	13.7		14	20	
	VP303 + *utx-1* RNAi	42	16.2	18% increase	16	22	0.008 (**)

Table S8. Statistical analysis of lifespan data relating to Figure 6

Full statistical analysis of lifespan data from Fig. 6 (****p<0.0001,***p<0.001,**p<0.01,*p<0.05, ns=not significant). EV = empty vector control. Rep = repeat. Mus = muscle, epi = epidermal, neu = neuronal, int = intestinal.
