# Supplementary material for: H3K27 modifiers regulate lifespan in C. elegans in a context-dependent manner
Source: BMC Biol. 2021 Mar 25;19:59. doi: 10.1186/s12915-021-00984-8 (PMC7995591; doi:10.1186/s12915-021-00984-8)
Supplement: Supplementary file 14 — Additional file 14: Figure S6. daf-16 dependence of lifespan extension due to tissue-specific knockdown or overexpression of utx-1. A-B: Lifespan assays were performed on animals tissue-specifically overexpressing utx-1 in the intestine (A) or neurons (B) subjected to daf-16 RNAi. Overexpression of utx-1 gave a moderate lifespan extension in both of these utx-1 overexpressing strains subjected to daf-16 RNAi (p=0.0001 (***) intestinal; p=0.0002 (***) neuronal), suggesting partial daf-16 independence in each case. N2 and daf-16 (RNAi) controls are shared between experiments A and B (as the experiments were performed as a large set) although the graphs are separated for clarity. C-F: Lifespan assays were performed on wild type and daf-16(mu86) animals subjected to global (C) as well as epidermis (D), neuron (E) and intestine-specific (F) knockdown of utx-1. Knockdown of utx-1 in the wild type background in all cases caused lifespan extension, whereas this was largely abrogated in daf-16(mu86) mutants. EV= Empty Vector control (i.e. worms fed HT115 bacteria transformed with L4440 RNAi vector lacking a genomic insert). See Additional file 15: Table S9 for full statistical analysis of lifespan data, including repeats. [file 12915_2021_984_MOESM14_ESM.pdf]

Fig. S6

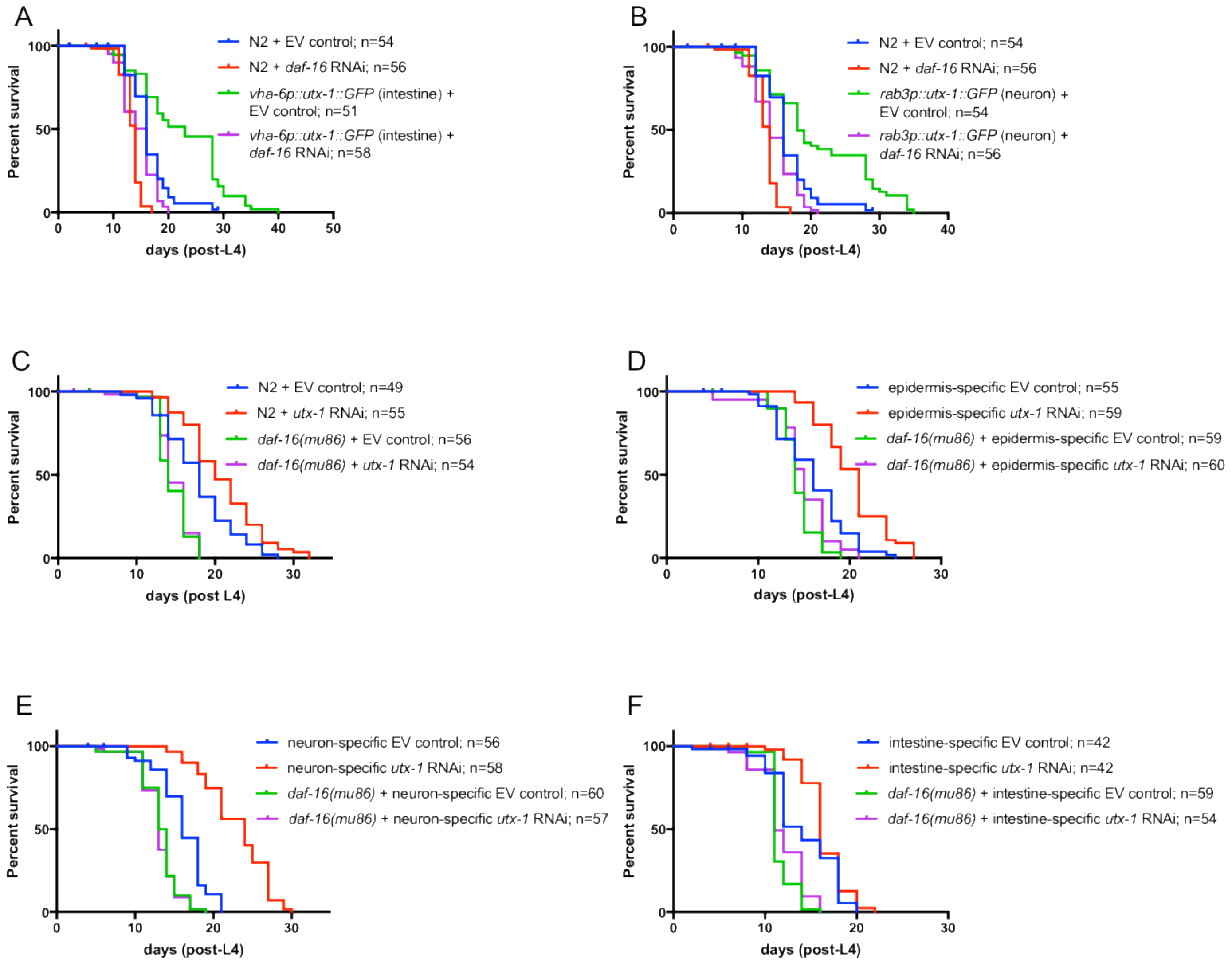

Figure S6. *daf-16* dependence of lifespan extension due to tissue-specific knockdown or overexpression of *utx-1*

A-B: Lifespan assays were performed on animals tissue-specifically overexpressing *utx-1* in the intestine (A) or neurons (B) subjected to *daf-16* RNAi. Overexpression of *utx-1* gave a moderate lifespan extension in both of these *utx-1* overexpressing strains subjected to *daf-16* RNAi (p=0.0001 (***) intestinal; p=0.0002 (***) neuronal), suggesting partial *daf-16* independence in each case. N2 and *daf-16* (RNAi) controls are shared between experiments A and B (as the experiments were performed as a large set) although the graphs are separated for clarity. C-F: Lifespan assays were performed on wild type and *daf-16(mu86)* animals subjected to global (C) as well as epidermis (D), neuron (E) and intestine-specific (F) knockdown of *utx-1*. Knockdown of *utx-1* in the wild type background in all cases caused lifespan extension, whereas this was

largely abrogated in *daf-16(mu86)* mutants. EV= Empty Vector control (*i.e.* worms fed HT115 bacteria transformed with L4440 RNAi vector lacking a genomic insert). See Additional file 15: Table S9 for full statistical analysis of lifespan data, including repeats.
