# Supplementary material for: H3K27 modifiers regulate lifespan in C. elegans in a context-dependent manner
Source: BMC Biol. 2021 Mar 25;19:59. doi: 10.1186/s12915-021-00984-8 (PMC7995591; doi:10.1186/s12915-021-00984-8)
Supplement: Supplementary file 15 — Additional file 15: Table S9. Statistical analysis of lifespan data relating to Figure S6. Full statistical analysis of lifespan data from Fig. S6 (****p<0.0001,***p<0.001,**p<0.01,*p<0.05, ns=not significant). EV = empty vector control. Rep = repeat. Note some of the data corresponds to data in Fig. 6 (Table S8) as these values were part of the same experiment, split off for clarity. [file 12915_2021_984_MOESM15_ESM.pdf]

Table S9

Fig ref	Strain / condition	no. of animals	mean lifespan	median lifespan	maximum lifespan	Log Rank Test *p* value relative to control
**S6A**	N2 EV control	54	16.3	16	29	
	N2 + *daf-16* RNAi	56	13.3	14	17	<0.0001 (****) compared with *N2 control*
	vha-6p::utx-1::GFP (intestine) + EV control	51	22.4	23	40	<0.0001 (****) compared with *N2 control*
	vha-6p::utx-1::GFP (intestine) + *daf-16* RNAi	58	14.47	16	20	0.0001 (***) compared with *daf-16 RNAi* <0.0001 (****) compared with *vha-6p::utx-1::GFP*
**S6A rep**	N2 EV control	57	16.3	18	22	
	N2 + *daf-16* RNAi	59	14.9	13	18	0.002 (**) compared with *N2 control*
	vha-6p::utx-1::GFP (intestine) + EV control	54	17.9	15	36	0.03 (*) compared with *N2 control*
	vha-6p::utx-1::GFP (intestine) + *daf-16* RNAi	57	15.51	13	26	0.03 (*) compared with *daf-16 RNAi* 0.008 (**) compared with *vha-6p::utx-1::GFP*
**S6B**	N2 EV control	54	16.3	16	29	
	N2 + *daf-16* RNAi	56	13.3	14	17	<0.0001 (****) compared with *N2 control*
	rab-3p::utx-1::GFP (neuron) + EV control	54	20.7	30	46	0.0002 (***) compared with *N2 control*
	rab-3p::utx-1::GFP (neuron) + *daf-16* RNAi	56	14.52	16	24	0.0002 (***) compared with *daf-16 RNAi* <0.0001 (****) compared with *utx-1(tm3118)+ utx-1::GFP*
**S6B rep**	N2 EV control	57	16.3	18	22	
	N2 + *daf-16* RNAi	59	14.9	13	18	0.002 (**) compared with *N2 control*
	rab-3p::utx-1::GFP (neuron) + EV control	53	18.7	19	37	0.001 (**) compared with *N2 control*
	rab-3p::utx-1::GFP (neuron) + *daf-16* RNAi	57	15.49	15	22	0.002 (**) compared with *daf-16 RNAi* 0.001 (**) *compared with utx-1(tm3118) +utx-1::GFP*

**S6C**	N2 + EV control	49	17.8	18	28	
	N2 + *utx-1* RNAi	55	20.8	20	32	0.003 (**) (vs N2 + EV)
	daf-16(mu86) + EV control	56	14.5	14	18	<0.0001 (****) (vs N2 + EV)
	daf-16(mu86) + *utx-1* RNAi	54	14.7	14	18	0.38 (ns) (vs daf-16(mu86)+EV)
**S6C rep**	N2 + EV control	54	14.4	14	20	
	N2 + *utx-1* RNAi	54	16.3	16	22	0.005 (**) (vs N2 + EV)
	daf-16(mu86) + EV control	56	14.3	14	17	0.002 (**) (vs N2 + EV)
	daf-16(mu86) + *utx-1* RNAi	57	14.7	14	17	0.27 (ns) (vs daf-16(mu86)+EV)
**S6D**	epidermis-specific EV control (strain NR222)	55	15.8	16	25	
	epidermis-specific *utx-1* RNAi	59	20.2	21	27	<0.0001 (****) (vs epidermis-specific EV)
	daf-16(mu86) + epidermis-specific EV control (strain AW1708)	59	14.3	16	22	0.0001 (***) (vs epidermis-specific EV)
	daf-16(mu86) + epidermis-specific *utx-1* RNAi	60	14.8	18	23	0.02 (*) (vs daf-16(mu86)+epidermis-specific EV)
**S6D rep**	epidermis-specific EV control (strain NR222)	44	14.4	14	24	
	epidermis-specific *utx-1* RNAi	43	19.4	20	28	<0.0001 (****) (vs epidermis-specific EV)
	daf-16(mu86) + epidermis-specific EV control (strain AW1708)	58	16.3	16	22	0.0001 (***) (vs epidermis-specific EV)
	daf-16(mu86) + epidermis-specific *utx-1* RNAi	58	18.3	18	23	0.0001 (***) (vs daf-16(mu86)+epidermis-specific EV)
**S6E**	neuron-specific EV control (strain TU3401)	56	16.1	16	21	
	neuron-specific *utx-1* RNAi	58	22.8	24	30	<0.0001 (****) (vs neuron-specific EV)
	daf-16(mu86) + neuron-specific EV control (strain AW1709)	60	13.3	13.5	19	<0.0001 (****) (vs neuron-specific EV)
	daf-16(mu86) + neuron-specific *utx-1* RNAi	57	13.0	13	19	0.57 (ns) (vs daf-16(mu86)+neuron-specific EV)
**S6E rep**	neuron-specific EV control (strain TU3401)	48	15.1	14	20	
	neuron-specific *utx-1* RNAi	48	21.8	22	29	<0.0001 (****) (vs neuron-specific EV)
	daf-16(mu86) + neuron-specific EV	57	12.8	14	17	<0.0001 (****) (vs neuron-specific EV)

	control (strain AW1709)					
	daf-16(mu86) + neuron-specific *utx-1* RNAi	58	13.0	14	16	0.79 (ns) (vs daf-16(mu86)+neuron-specific EV)
**S6F**	intestine-specific EV control (strain VP303)	42	13.7	14	20	
	intestine-specific *utx-1* RNAi	42	16.2	16	22	0.008 (**) (vs intestine-specific EV)
	daf-16(mu86) + intestine-specific EV control (strain AW1774)	59	11.6	11	14	<0.0001 (****) (vs intestine-specific-EV)
	daf-16(mu86) + intestine-specific *utx-1* RNAi	54	11.9	11	14	0.07 (ns) (vs daf-16(mu86)+intestine-specific EV)
**S6F rep**	intestine-specific EV control (strain VP303)	51	14.7	14	24	
	intestine-specific *utx-1* RNAi	56	16.6	16	24	0.009 (**) (vs intestine-specific-EV)
	daf-16(mu86) + intestine-specific EV control (strain AW1774)	56	12.5	12	16	0.0003 (***) (vs intestine-specific-EV)
	daf-16(mu86) + intestine-specific *utx-1* RNAi	55	12.4	11	16	0.76 (ns) (vs daf-16(mu86)+intestine-specific EV)

Table S9. Statistical analysis of lifespan data relating to Figure S6

Full statistical analysis of lifespan data from Fig. S6 (****p<0.0001,***p<0.001,**p<0.01,*p<0.05, ns=not significant). EV = empty vector control. Rep = repeat. Note some of the data corresponds to data in Figure 6 (Table S8) as these values were part of the same experiment, split off for clarity.
