# Supplementary material for: H3K27 modifiers regulate lifespan in C. elegans in a context-dependent manner
Source: BMC Biol. 2021 Mar 25;19:59. doi: 10.1186/s12915-021-00984-8 (PMC7995591; doi:10.1186/s12915-021-00984-8)
Supplement: Supplementary file 16 — Additional file 16: Figure S7. Knockdown of daf-2 enhances longevity in a tissue-specific manner. A: Global depletion of daf-2 was achieved through RNAi and promoted lifespan extension in wild-type animals compared to EV controls (p<0.0001 (****)). B-E: Tissue-specific depletion of daf-2 enhanced worm longevity when knocked down in the epidermis (B), intestine (C) and neurons (D) (p<0.0001 (****) in all cases), but not in the muscle (E) (p=0.82 (ns)), the same tissues implicated in improved longevity mediated by utx-1 knockdown. EV= Empty Vector control (i.e. worms fed HT115 bacteria transformed with L4440 RNAi vector lacking a genomic insert). See Additional file 17: Table S10 for full statistical analysis of lifespan data. [file 12915_2021_984_MOESM16_ESM.pdf]

Fig. S7

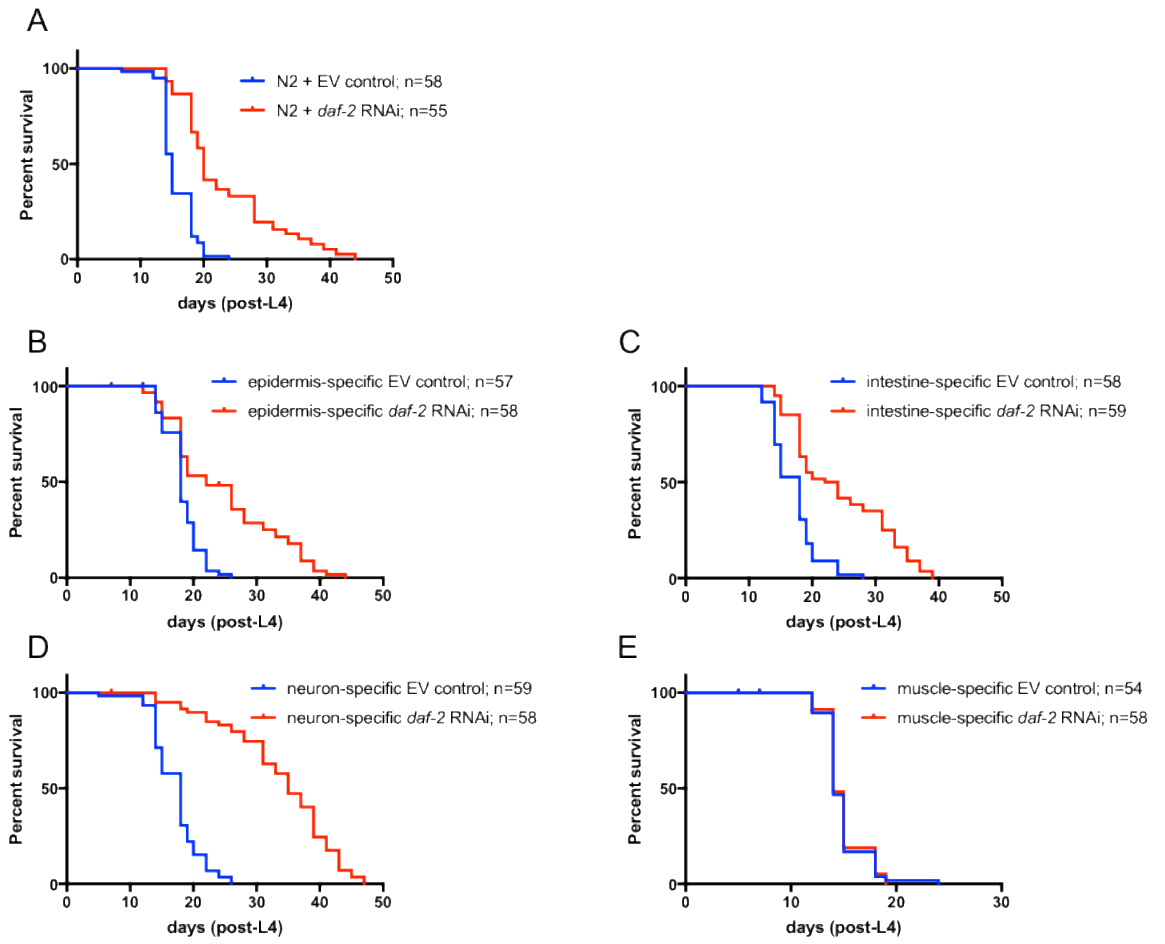

Figure S7. Knockdown of *daf-2* enhances longevity in a tissue-specific manner

A: Global depletion of *daf-2* was achieved through RNAi and promoted lifespan extension in wild-type animals compared to EV controls ($p<0.0001$ (****)). B-E: Tissue-specific depletion of *daf-2* enhanced worm longevity when knocked down in the epidermis (B), intestine (C) and neurons (D) ($p<0.0001$ (****) in all cases), but not in the muscle (E) ($p=0.82$ (ns)), the same tissues implicated in improved longevity mediated by *utx-1* knockdown. EV= Empty Vector control (*i.e.* worms fed HT115 bacteria transformed with L4440 RNAi vector lacking a genomic insert). See Additional file 17: Table S10 for full statistical analysis of lifespan data.
