# Supplementary material for: H3K27 modifiers regulate lifespan in C. elegans in a context-dependent manner
Source: BMC Biol. 2021 Mar 25;19:59. doi: 10.1186/s12915-021-00984-8 (PMC7995591; doi:10.1186/s12915-021-00984-8)
Supplement: Supplementary file 17 — Additional file 17: Table S10. Statistical analysis of lifespan data relating to Figure S7. Full statistical analysis of lifespan data from Fig. S7 (****p<0.0001,***p<0.001,**p<0.01,*p<0.05, ns=not significant). EV = empty vector control. Epi = epidermal, int = intestinal neu = neuronal, mus = muscle. [file 12915_2021_984_MOESM17_ESM.pdf]

Table S10

Fig ref	Strain / condition	no. of animals	mean lifespan	% lifespan change (vs control)	median lifespan	maximum lifespan	Log Rank Test *p* value relative to control
**S7A**	N2 + EV RNAi	58	15.7		15	24	
	N2 + *daf-2* RNAi	55	22.3	42% increase	20	44	<0.0001 (****)
**S7B**	epidermis-specific EV control (strain: NR222)	57	18.2		18	26	
**(epi)**	epidermis-specific *daf-2* RNAi	58	24.4	34% increase	22	44	<0.0001 (****)
**S7C**	intestine-specific EV control (strain: VP303)	58	16.7		18	28	
**(int)**	intestine-specific *daf-2* RNAi	59	24.3	46% increase	23	39	<0.0001 (****)
**S7D**	neuron-specific EV control (strain: TU3401)	59	17.2		18	26	
**(neu)**	neuron-specific *daf-2* RNAi	58	33.7	96% increase	35	47	<0.0001 (****)
**S7E**	muscle-specific EV control (strain: NR350)	54	14.9		14	24	
**(mus)**	muscle-specific *daf-2* RNAi	58	14.9		14	19	0.82 (ns)

Table S10. Statistical analysis of lifespan data relating to Figure S7. Full statistical analysis of lifespan data from Fig. S7 (****$p<0.0001$,***$p<0.001$,**$p<0.01$,*$p<0.05$, ns=not significant). EV = empty vector control. Epi = epidermal, int = intestinal neu = neuronal, mus = muscle.
