# Supplementary material for: H3K27 modifiers regulate lifespan in C. elegans in a context-dependent manner
Source: BMC Biol. 2021 Mar 25;19:59. doi: 10.1186/s12915-021-00984-8 (PMC7995591; doi:10.1186/s12915-021-00984-8)
Supplement: Supplementary file 18 — Additional file 18: Table S11. List of C. elegans strains used in this study. Strain names are indicated along with genotypes, plasmids and short names used in this report. [file 12915_2021_984_MOESM18_ESM.pdf]

Table S11

Strain name	Genotype	Strain name used for the purpose of this study
N2		wild type (WT)
	mes-2(tm5007) II	*mes-2(tm5007)*
SS186	*mes-2(bn11) unc-4(e120)/mnC1 dpy-10(e128) unc-52(e444) II*	*mes-2(bn11)*
VC2409	*mes-2(ok2480)/mT1 II; +/mT1 [dpy-10(e128)] III*	*mes-2(ok2480)*
	jmjd-3.2(tm3121) X	*jmjd-3.2(tm3121)*
ZR332	*jmjd-3.1(gk384) jmjd-3.2 (tm3121) jmjd-3.3(tm3197) X*	*jmjd-3* triple mutant
ZR252	*utx-1(tm3118) X*	*utx-1(tm3118)*
ZR254<br>ZR255	*utx-1(tm3118) X ; zrEx264[utx-1p::utx-1::GFP + rol-6^{+}]*	*utx-1(tm3118)+ utx-1 OE*
ZR256<br>ZR257	*utx-1(tm3118) X; zrEx295[utx-1p::utx-1DD::GFP + rol-6^{+}]*	*utx-1(tm3118)+ utx-1DD OE*
ZR802	*zrEx264[utx-1p::utx-1::GFP + rol-6^{+}]*	*utx-1 OE*
ZK856	*zrEx295[utx-1p::utx-1DD::GFP + rol-6^{+}]*	*utx-1DD OE*
ZR693<br>ZR694	*zrEx231[myo-3p::utx-1::GFP + rol-6^{+} + myo-2::rfp]*	*myo-3p::utx-1:: GFP*
ZR695<br>ZR696	*zrEx233[dpy-7p::utx-1::GFP + rol-6^{+} + myo-2::rfp]*	*dpy-7p::utx-1:: GFP*
ZR697<br>ZR698	*zrEx235[rab-3p::utx-1::GFP + rol-6^{+} + myo-2::rfp]*	*rab-3p::utx-1:: GFP*
ZR699<br>ZR700	*zrEx237[vha-6p::utx-1::GFP + rol-6^{+} + myo-2::rfp]*	*vha-6p::utx-1:: GFP*
ZR701<br>ZR702	*jmjd-3.2(tm3121) X zrEx239[jmjd-3.2p::jmjd-3.2::GFP + rol-6^{+} + myo-2::rfp]*	*jmjd-3.2(tm3121)+ jmjd-3.2 OE*
ZR703<br>ZR704	*jmjd-3.2(tm3121) X zrEx241[jmjd-3.2p::jmjd3.2DD::GFP + rol-6^{+} + myo-2::rfp]*	*jmjd-3.2(tm3121) + jmjd-3.2DD OE*
ZR705	*zrEx243[jmjd-3.2p::jmjd-3.2::GFP + rol-6^{+} +myo-2::rfp]*	*jmjd-3.2 OE*
ZR706<br>ZR707	*zrEx245[jmjd-3.2p::jmjd-3.2DD::GFP + rol-6^{+} + myo-2::rfp]*	*jmjd-3.2DD OE*
VP303	*rde-1(ne219) V; kbIs7 [nhx-2p::rde-1 + rol-6(su1006)]*	intestine specific RNAi
NR222	*rde-1(ne219) V; kzIs9 [(pKK1260) lin-26p::NLS::GFP + (pKK1253) lin-26p::rde-1 + rol-6(su1006)]*	epidermis specific RNAi

NR350	*rde-1(ne219) V; kzIs20 [hlh-1p::rde-1 + sur-5p::NLS::GFP]*	muscle specific RNAi
TU3401	*sid-1(pk3321) V; uIs69 [pCFJ90 (myo-2p::mCherry) + unc-119p::sid-1]*	neuronal specific RNAi
AW1708	*daf-16(mu86) I; rde-1(ne219) V; kzIs9 [(pKK1260) lin-26p::NLS::GFP + (pKK1253) lin-26p::rde-1 + rol-6(su1006)]*	*daf-16(mu86); epidermis-specific RNAi*
AW1709	*daf-16(mu86) I; sid-1(pk3321) V; uIs69 [pCFJ90 (myo-2p::mCherry) + unc-119p::sid-1]*	*daf-16(mu86); neuron-specific RNAi*
AW1774	*daf-16(mu86) I; rde-1(ne219) V; kbIs7 [nhx-2p::rde-1 + rol-6(su1006)]*	*daf-16(mu86); intestine-specific RNAi*
TJ356	*zIs356* [*daf-16p::daf-16a/b::GFP + rol-6(su1006)*]	*daf-16::GFP translational reporter*

Table S11. List of *C. elegans* strains used in this study. Strain names are indicated along with genotypes, plasmids and short names used in this report.
